# Correlated receptor transport processes buffer single-cell heterogeneity

**DOI:** 10.1371/journal.pcbi.1005779

**Published:** 2017-09-25

**Authors:** Stefan M. Kallenberger, Anne L. Unger, Stefan Legewie, Konstantinos Lymperopoulos, Ursula Klingmüller, Roland Eils, Dirk-Peter Herten

**Affiliations:** 1 Department for Bioinformatics and Functional Genomics, Division of Theoretical Bioinformatics, German Cancer Research Center (DKFZ), Institute for Pharmacy and Molecular Biotechnology (IPMB) and BioQuant, Heidelberg University, Heidelberg, Germany; 2 Cellnetworks Cluster and Institute of Physical Chemistry, BioQuant, Heidelberg University, Heidelberg, Germany; 3 Institute of Molecular Biology, Mainz, Germany; 4 Division Systems Biology of Signal Transduction, DKFZ-ZMBH Alliance, German Cancer Research Center (DKFZ), Heidelberg, Germany; 5 Translational Lung Research Center (TLRC), Member of the German Center for Lung Research (DZL), Heidelberg, Germany; Johns Hopkins University, UNITED STATES

## Abstract

Cells typically vary in their response to extracellular ligands. Receptor transport processes modulate ligand-receptor induced signal transduction and impact the variability in cellular responses. Here, we quantitatively characterized cellular variability in erythropoietin receptor (EpoR) trafficking at the single-cell level based on live-cell imaging and mathematical modeling. Using ensembles of single-cell mathematical models reduced parameter uncertainties and showed that rapid EpoR turnover, transport of internalized EpoR back to the plasma membrane, and degradation of Epo-EpoR complexes were essential for receptor trafficking. EpoR trafficking dynamics in adherent H838 lung cancer cells closely resembled the dynamics previously characterized by mathematical modeling in suspension cells, indicating that dynamic properties of the EpoR system are widely conserved. Receptor transport processes differed by one order of magnitude between individual cells. However, the concentration of activated Epo-EpoR complexes was less variable due to the correlated kinetics of opposing transport processes acting as a buffering system.

## Author summary

Cell surface receptors translate extracellular ligand concentrations to intracellular responses. Receptor transport between the plasma membrane and other cellular compartments regulates the number of accessible receptors at the plasma membrane that determines the strength of downstream pathway activation at a given ligand concentration. In cell populations, pathway activation strength and cellular responses vary between cells. Understanding origins of cell-to-cell variability is highly relevant for cancer research, motivated by the problem of fractional killing by chemotherapies and development of resistance in subpopulations of tumor cells. The erythropoietin receptor (EpoR) is a characteristic example of a receptor system that strongly depends on receptor transport processes. It is involved in several cellular processes, such as differentiation or proliferation, regulates the renewal of erythrocytes, and is expressed in several tumors. To investigate the involvement of receptor transport processes in cell-to-cell variability, we quantitatively characterized trafficking of EpoR in individual cells by combining live-cell imaging with mathematical modeling. Thereby, we found that EpoR dynamics was strongly dependent on rapid receptor transport and turnover. Interestingly, although transport processes largely differed between individual cells, receptor concentrations in cellular compartments were robust to variability in trafficking processes due to the correlated kinetics of opposing transport processes.

## Introduction

In cells external signals from ligands are transmitted by receptors to intracellular signaling cascades. Receptor signaling is regulated by receptor transport processes between the plasma membrane and other cellular compartments that are subsumed under the term receptor trafficking [1]. In absence of ligand, receptors are transported to the plasma membrane and are taken up again by the cell. After ligand binding, activated receptors at the plasma membrane can be internalized. To shut down signal transduction, endosomal acidification induces ligand dissociation from the receptor. Subsequently, the receptor is either degraded or transported back to the plasma membrane. These transport processes therefore strongly influence the ability of cells to integrate signals from external ligands and thereby the translation into cellular responses.

In a variety of receptor systems, receptor trafficking was quantitatively studied by a combination of experiments and ODE models based on population average data [2–4]. For example, endocytosis, degradation and receptor recycling were quantitatively studied in the epidermal growth factor receptor (EGFR) [5–10], the erythropoietin (Epo) receptor [11,12], the insulin receptor [13,14], chemotactic peptide receptors on neutrophils [15–17], the transferrin receptor (TfR) [18,19], the low density lipoprotein receptor (LDLR) [20,21], interferon-α and tumor necrosis factor receptors [22,23]. These studies established a canonical receptor trafficking model that accounts for exchange of free receptors between the plasma membrane compartment and an intracellular receptor pool, internalization of ligand-bound receptors, degradation, and receptor recycling [2–4,24]. Quantifying receptor trafficking processes helped to characterize physiologically relevant differences between receptor systems. In particular, kinetic parameters for ligand binding, the internalization of free or ligand-bound receptors and for synthesis and degradation of receptors showed large differences between receptor systems, and could be used to categorize receptors according to functional roles in cells [2,4,24]. Growth factor receptors such as the EGFR are characterized by a high membrane abundance and a strongly accelerated internalization of ligand-bound compared to free receptors at the plasma membrane, a phenomenon denoted as ligand-induced receptor downregulation [5,15,25]. Due to an accelerated internalization upon ligand binding, short reaction times of receptor signaling to changes in ligand concentrations are facilitated [24]. From a systems perspective, this increases the accuracy of signal transduction within involved signaling pathways [4,24]. On the contrary, transport receptors as the TfR or the LDLR typically do not exhibit an accelerated internalization upon ligand binding but show a high rate of receptor internalization compared to the rate of ligand unbinding [24,26–28]. Cytokine receptors, as the EpoR or the interleukin 3 receptor, are characterized by a low membrane abundance and an efficient clearance of ligand from the medium and rapid recovery of receptor levels at the plasma membrane [4,12].

The last four decades contributed to a broad understanding of dynamic properties of receptor systems but most studies described receptor trafficking based on measurements of cell population averages. Because trafficking processes depend on a multitude of biochemical processes including for example vesicle formation and cytoskeleton-dependent transport [1,29], heterogeneous expression of involved proteins can give rise to cell-to-cell variability [30]. In this context, an open question is whether cellular heterogeneity in different receptor trafficking processes can dissolve borders between categories of receptor systems, potentially leading to subpopulations of cells showing features as endocytic downregulation, fast replenishment or an efficient receptor recycling. As a result, cell-to-cell variability in receptor trafficking might cause a diverging behavior of cells in response to an external stimulus. For this reason, it is an important question whether receptor systems exhibit robustness to cellular variability in trafficking processes. A prime example for the importance of receptor transport processes in regulating systems properties is the receptor for the hormone erythropoietin (Epo) [11]. Ligand-induced signal transduction through this cytokine receptor, the EpoR, comprises primarily activation of JAK2/STAT5, PI3K/AKT and MAPK pathways, and is absolutely essential for differentiation, proliferation and cell survival of erythroid progenitor cells to ensure renewal of mature erythrocytes [31,32]. Transport processes regulating EpoR induced signal transduction are (i) receptor internalization and inactivation followed by subsequent degradation, and (ii) receptor recycling encompassing ligand-induced receptor endocytosis and subsequent transport back to the plasma membrane [12,33]. It was reported that the activation of kinases and phosphatases [34], ubiquitination of the receptor [35], and cargo protein and cytoskeleton dependent processes such as assembly of actin oligomers [36] modulate transport of the EpoR.

A characteristic property of the EpoR system is that only a small fraction of the total receptor amount is present at the cell surface [37,38]. By dynamic pathway modeling in combination with binding studies utilizing radioactively labeled Epo we recently showed that extremely rapid receptor turn-over ensures responsiveness of the system for a very broad ligand-concentration range as it is for example observed during continuous erythrocyte renewal and accelerated production in response to severe blood loss [11,12,39]. Further, data-based mathematical models revealed that (1) Epo-induced activation of the JAK2-STAT5 signaling cascade occurs in cycles continuously monitoring the activation status of the receptor [11,12,39] and (2) the two induced negative regulators bind to the receptor and divide the labor to control signaling for a wide range of Epo concentrations [31,32]. The so far established mathematical models were calibrated based on cell population data obtained for suspension cells. The kinetics at the level of single cells is smoothed and underlying biochemical signaling networks might be misinterpreted due to averaging population heterogeneities [40–42]. Furthermore, since the EpoR is also expressed on some tumor cells such as non-small cell lung carcinoma cell lines [43], it of much interest to investigate to which degree principles learned in suspension cells can be transferred to adherent cancer cells.

Here, we developed an approach based on live cell imaging, image segmentation of subcellular compartments, and cell ensemble models to investigate the extent of variability in receptor trafficking and interrelations between the dynamics of transport processes. Single-cell measurements of EpoR concentrations in different cellular compartments were used to estimate kinetic parameters of receptor trafficking processes for individual cells. By model discrimination we determined which receptor transport processes essentially contributed to receptor trafficking of EpoR. Calibrating cell ensemble models with a combination of single-cell datasets improved the identifiability of single-cell kinetic parameters, which was a prerequisite for analyzing correlations between kinetic parameters of receptor transport processes. Despite the large variability in the EpoR trafficking reactions we observed that the correlation between the kinetics of different transport processes had a buffering effect on the concentration of Epo-EpoR complexes at the plasma membrane and in the endosomal compartment. This correlation of the kinetics of different processes involved in the same cellular signaling system might represent a general motif of biological systems to confine cell-to-cell variability.

## Results

### Quantification of EpoR transport processes in single cells

**Fig 1:**
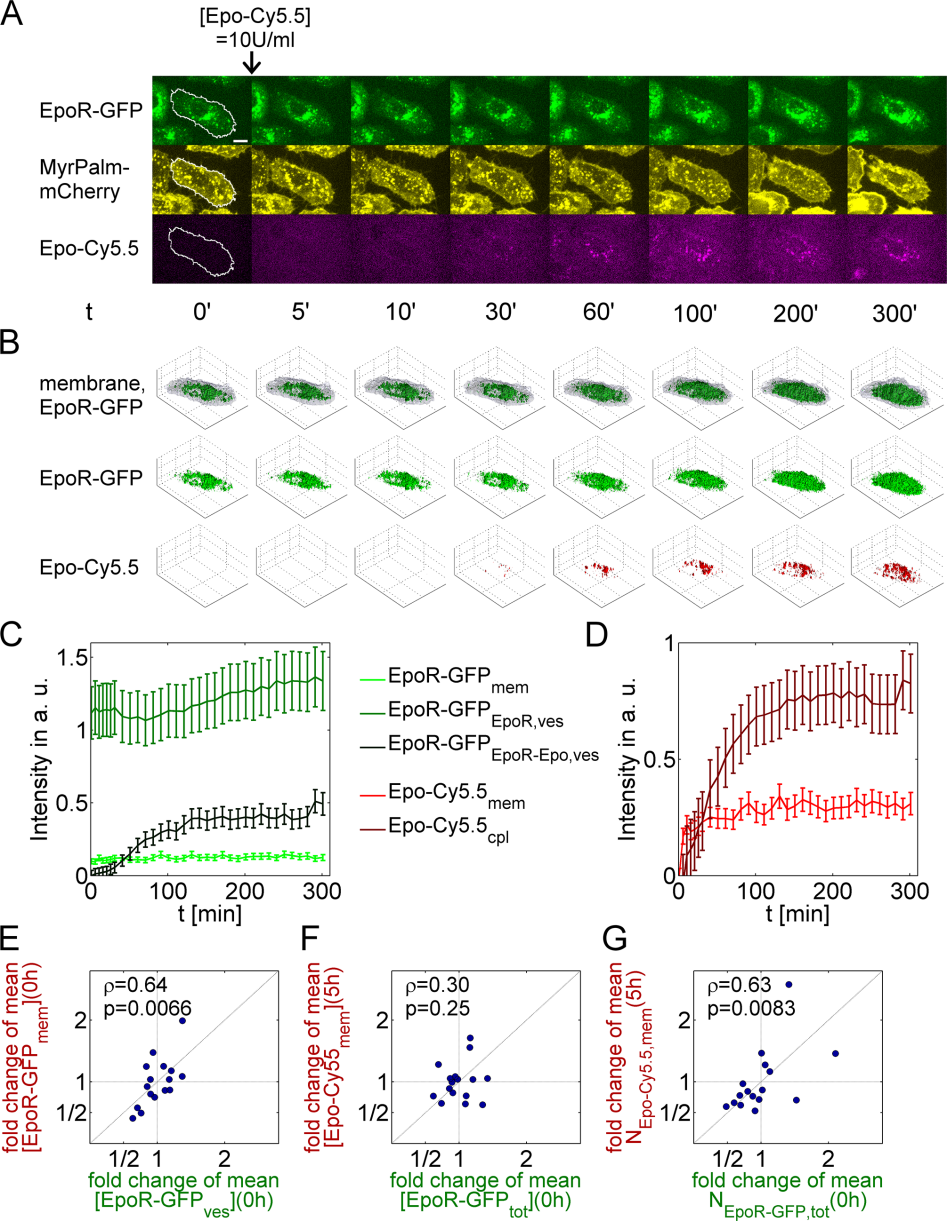
Single-cell quantification of EpoR-GFP and Epo-Cy5.5 internalization. **(A)** Single planes of 3D stack images of an exemplary H838 cells for EpoR-GFP (green), MyrPalm-mCherry (yellow) and Epo-Cy5.5 (purple) are shown for different time points. Membrane ROIs were defined in each plane by cellular outlines detected in MyrPalm-mCherry images, as indicated by the white lines at t = 0’ (MyrPalm, myristoylation-palmitoylation domain, scale bar: 10μm). After the first time point, 10U/ml Epo-Cy5.5 was added. **(B)** Rendering of EpoR-GFP containing vesicles and membrane ROIs as transparent overlay (first row), EpoR-GFP containing vesicles alone (second row) and Epo-Cy5.5 containing vesicles (third row) for the cell shown in panel A. **(C)** Single-cell GFP fluorescence intensity trajectories in the membrane ROI (EpoR-GFP_mem_), in vesicles containing only EpoR-GFP (EpoR-GFP_EpoR,ves_) or EpoR-GFP and Epo-Cy5.5 (EpoR-GFP_EpoR-Epo,ves_) after normalization to the cell volume for the cell in (A) and (B). **(D)** Cy5.5 fluorescence in the membrane ROI (Epo-Cy5.5_mem_) and in the whole cytoplasm (Epo-Cy5.5_ves_) normalized to the cell volume. **(E–G)** Correlations between fold changes (circles) of single-cell EpoR-GFP or Epo-Cy5.5 concentrations or absolute amounts, individually quantified for H838 cells, relative to population averages (ρ, Pearson correlation coefficient, p-values obtained from t-tests). (E) Correlation between EpoR-GFP concentrations in the plasma membrane ROI ([EpoR-GFP_mem_]) at 0h, and of EpoR-GFP concentrations in intracellular vesicles ([EpoR-GFP_ves_]) at 0h. (F) Correlation between single-cell concentrations of Epo-Cy5.5 ([Epo-Cy5.5_mem_]) at 5h and total cellular EpoR-GFP concentrations ([EpoR-GFP_tot_]) at 0h. (G) Correlation between single-cell absolute molecule numbers of Epo-Cy5.5 (N_Epo-Cy5.5,mem_) at 5h and total cellular EpoR-GFP amounts (N_EpoR-GFP,tot_) at 0h.

The EpoR is transported to the plasma membrane, can bind Epo, and is subjected to endocytosis, degradation and transport back to the plasma membrane [12,33]. To quantitatively study these processes at the single-cell level, we developed an approach employing an EpoR-GFP fusion protein (EpoR-GFP) and Epo labeled with the organic dye Cy5.5 (Epo-Cy5.5). The EpoR-GFP fusion protein was stably expressed in the NSCLC cell line H838 and a fluorescent membrane marker, mCherry fused to a myristoylation-palmitoylation (MyrPalm) domain (MyrPalm-mCherry) accumulating at the plasma membrane was co-expressed (Fig 1A) [44]. After recording the first image stack, cells were exposed to Epo-Cy5.5 at a concentration of 4.2nM corresponding to a biological activity of 10U/ml Epo [45]. Subsequently, Epo internalization was studied for at least five hours by recording three-dimensional stacks of confocal microscope images. Analyzing Epo-Cy5.5 in combination with EpoR-GFP and the membrane marker MyrPalm-mCherry enabled simultaneous recording of complementary information on Epo-uptake, EpoR-internalization and EpoR-degradation essential for studying protein turnover by kinetic modeling. While the GFP signal indicated the amount of EpoR-GFP and was affected by EpoR-GFP degradation, the Cy5.5 signal represented the sum of intact and degraded proteins since the dye molecule Cy5.5 is not targeted by protein degradation mechanisms.

Intensities for membrane and cytosolic compartments were extracted from microscopic data to obtain time-resolved measurements, which were proportional to local concentrations of EpoR-GFP and Epo-Cy5.5, and were used for model fitting. For this purpose, we developed a segmentation software to semi-automatically define three-dimensional regions of interest (ROIs) for the plasma membrane using the MyrPalm-mCherry signal, and for EpoR-GFP/Epo-Cy5.5 containing vesicles the EpoR-GFP and Epo-Cy5.5 signals (Fig 1A to 1C, S1 Fig and S1 Movie; for details, see S1 Text). In the ROI for the plasma membrane, Epo-Cy5.5 intensities were associated with the amount of Epo-EpoR complexes, while EpoR-GFP intensities were associated with the total amount of EpoR (Epo-ligated plus free receptors). Further, based on an intensity threshold for Cy5.5, we distinguished between EpoR in voxels containing only EpoR-GFP or Epo-Cy5.5/EpoR-GFP (S1 Text). We extracted the Cy5.5 fluorescence signal in the cytosolic compartment to obtain a quantitative measure of the amount of internalized Epo-Cy5.5. Epo that was bound to internalized EpoR can be either secreted from the cell or degraded. Because Cy5.5 that was coupled to Epo is not proteolytically degraded, the intracellular Cy5.5 signal was assumed to reflect the amount of intact and degraded Epo. To obtain quantities for model fitting that were proportional to EpoR and Epo concentrations, intensities were normalized by cellular volumes, which were defined by the volumes enclosed by outer borders of membrane ROIs.

The described procedure was applied to analyze for example 16 single Epo-treated H838 cells. As shown in Fig 1C for a representative single cell, we observed the strongest signal changes within the first hour after addition of Epo-Cy5.5, indicating fast binding and internalization. The membrane EpoR-GFP fraction and the signal from EpoR-GFP vesicles showed in the exemplary cell only a slight increase, implying that Epo did not have a large influence on the total amount of the EpoR. On the contrary, the intensity from Epo-Cy5.5-containing vesicles continuously increased. While the Cy5.5 intensity at the plasma membrane reached a steady state after about ten minutes, intracellular Cy5.5 intensity showed a prolonged increase suggesting a slow decay of internalized Epo-Cy5.5.

We asked whether initial conditions such as EpoR concentrations in cellular compartments were predictive for EpoR trafficking in the presence of Epo, and evaluated associations between characteristic measures of single-cell trajectories before and after adding Epo-Cy5.5. In particular, we examined which experimental quantities were predictive for the amount of membrane bound Epo-Cy5.5, which can be assumed to reflect the amount of active EpoR [11,12]. For all Epo-treated cells, characteristic parameters were extracted from segmented imaging data, resulting in total EpoR concentrations or EpoR numbers in arbitrary units. Absolute numbers of EpoR-GFP or Epo-Cy5.5 in cellular compartments were estimated by summing up fluorescence intensities in segmented compartment ROIs, while cellular concentrations were estimated by dividing fluorescence intensity sums in cellular compartment ROIs by cell volumes. For scale-free comparisons, single-cell measures were divided by the means of all cells to obtain fold changes relative to single-cell averages (Fig 1E–1G, S2 Fig). Among all cells, the membrane EpoR-GFP fraction contained on average 7.6% (SD: 2.1%) of the total cellular amount of EpoR-GFP. Interestingly, EpoR concentrations in the membrane ROI ([EpoR-GFP_mem_]) were significantly correlated with EpoR concentrations in intracellular vesicles ([EpoR-GFP_ves_]; Fig 1E; p = 0.0066 for Pearson correlation coefficients). This implies that the kinetics of EpoR transport from the cytosol to the plasma membrane was correlated with kinetics of EpoR transport from the plasma membrane back to the cytosol. The observation of correlated trafficking parameters will be further addressed below. Furthermore, while there was no significant correlation between the total cellular concentrations of EpoR-GFP ([EpoR-GFP_tot_]) and the concentration of Epo-Cy5.5 in the plasma membrane ROI ([Epo-Cy5.5_mem_]) at the end of the experiment after 5 hours (Fig 1F; p = 0.25), absolute amounts of cellular EpoR-GFP (N_EpoR-GFP,tot_) were significantly correlated with the amounts of membrane Epo-Cy5.5 (N_Epo-Cy5.5,mem_) at 5h (Fig 1G; p = 0.0083). While total amounts of EpoR-GFP and internalized Epo-Cy5.5 were significantly correlated, there were no significant correlations between EpoR-GFP and Epo-Cy5.5 concentrations in different cell compartments (S2 Fig), which indicates that the EpoR transport kinetics strongly varied between cells.

Taken together, we established an experimental setup to quantitatively study the dynamics of the EpoR and the internalization of Epo by live-cell microscopy.

### Discrimination between relevant and irrelevant EpoR transport processes by cell ensemble modeling

To mechanistically study cell-to-cell variability in EpoR transport processes, we developed different mathematical models (EpoR model) based on ordinary differential equations (ODE) and estimated the model parameters by model fitting to single-cell measurements. The EpoR model variants, consisting of a basic model and variable extensions, described the two observed species, free EpoR and EpoR bound to Epo, in different cellular compartments or at the plasma membrane.

**Fig 2:**
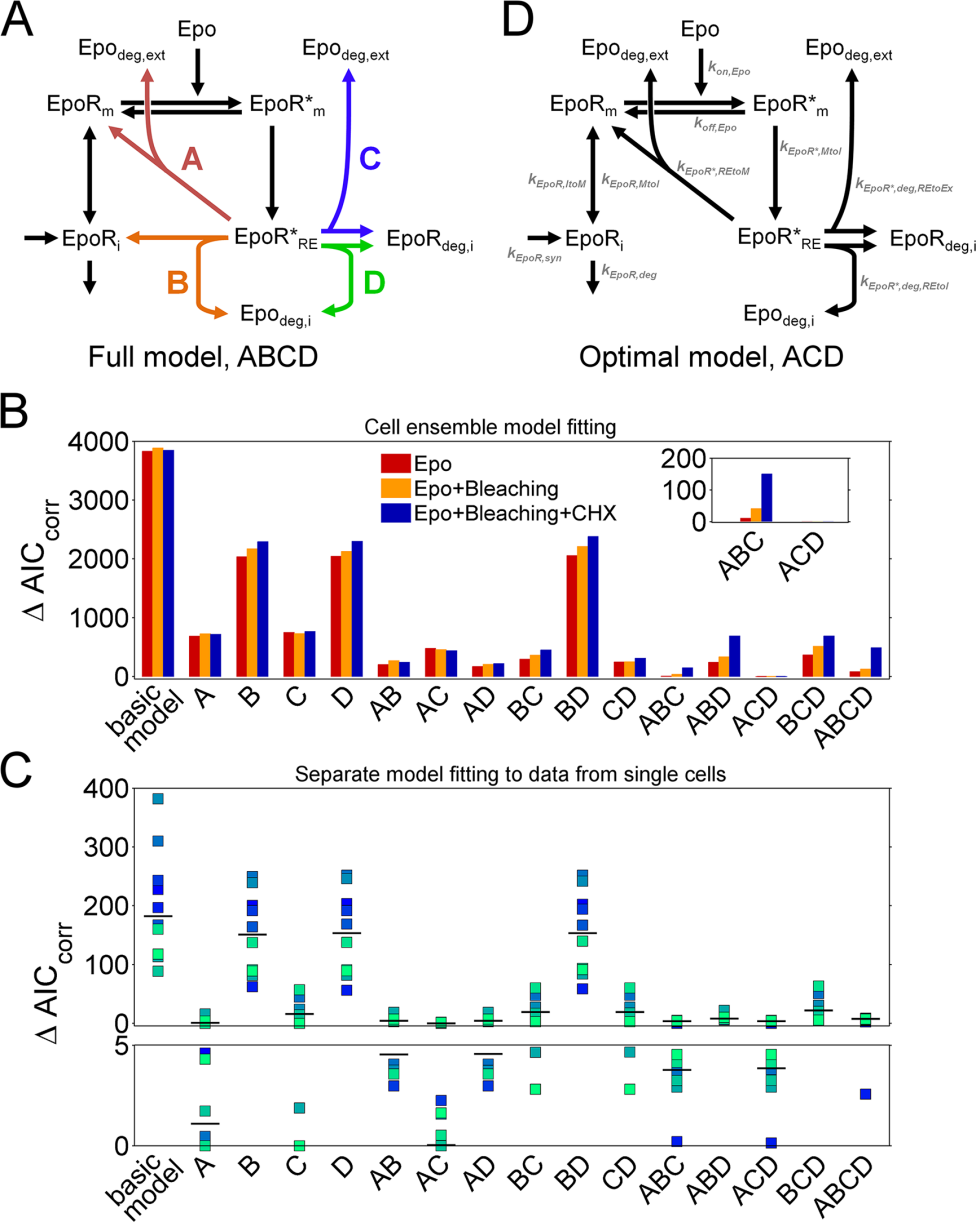
The optimal model variant contains reactions for EpoR recycling and degradation. **(A)** Basic model describing transport between membrane and intracellular receptors (EpoR_m_, EpoR_i_), reversible binding of Epo to EpoR*_m_ and internalization to recycling endosomal EpoR*_RE_ (black arrows), extended by variable parts A to D (A, direct EpoR recycling to the plasma membrane; B, recycling to intracellular pool and intracellular accumulation of degraded Epo; C, degradation and transport of degraded Epo_deg,ext_ to extracellular space; D, degradation and intracellular accumulation of degraded Epo_deg,i_). **(B)** Differences in AIC_corr_ values to the optimal model variant ACD for all variants, indicated for model fitting to data from only Epo internalizing cells (Epo), additionally bleached cells (Epo+Bleaching), and additionally CHX treated cells (Epo+Bleaching+CHX). The inlay shows that AIC_corr_ values for the ACD variant were clearly lower than for the next better model variant “ABC”. **(C)** Differences in AIC_corr_ to the optimal model variant for model fitting to data from only a single cell at a time, for ten selected cells, indicated by colors (squares, ΔAIC_corr_ values; black bars, median ΔAIC_corr_ values for each model variant). Notably, when fitting single-cell models to data from different individual cells, different model variants were optimal. **(D)** Topology of the optimal variant ACD and indication of kinetic parameters for EpoR trafficking reactions (global parameters: k_on,Epo_, k_off,Epo_, Epo binding and unbinding; single-cell parameters: k_EpoR,syn_, k_EpoR,deg_, EpoR turnover; k_EpoR,ItoM_, k_EpoR,MtoI_, transport between intracellular and plasma membrane compartments; k_EpoR*,MtoI_; endocytosis of Epo-ligated EpoR; k_EpoR*,REtoM_; recycling to plasma membrane; k_EpoR*,deg,REtoEx_ and k_EpoR*,deg,REtoI_, EpoR degradation with exocytosis or with intracellular accumulation of degraded Epo).

The basic EpoR model describes reversible binding of Epo to the EpoR at the plasma membrane (EpoR_m_) and formation of active EpoR (EpoR_m_*) (black arrows in Fig 2A). EpoR permanently cycle between the plasma membrane (EpoR_m_) and the intracellular compartment (EpoR_i_). The intracellular pool of the EpoR is subject to degradation and refilled by synthesis. Active EpoR at the membrane EpoR_m_* are internalized to the endocytic recycling compartment (EpoR_RE_*). In the model reaction describing EpoR binding to free Epo, Epo is not consumed because the amount of Epo in the medium largely exceeds the total amount of EpoR, as described in the methods section, and can therefore be assumed to remain constant. The basic model was extended by variable parts A to D, which described different possible ways for EpoR transport back to the plasma membrane or degradation. By appending variable combinations of parts A to D to the basic model, 16 possible model variants were formulated to systematically test the contribution of different processes to EpoR trafficking in our cellular system. Since receptor recycling and degradation of ligand-bound receptors were described for several receptor systems as the EGFR, IL3R or TfR [2,12,46–49], we explored their role in EpoR trafficking in our cellular system, and whether their contribution was essential or could be neglected, which was not examined in previous modeling studies on EpoR trafficking. In model variants, internalized Epo is either released back into the extracellular space (parts A and C) or degraded (Epo_deg,i_) and accumulates inside the cell (parts B and D, Fig 2A) [11,12]. After internalization of Epo-EpoR complexes, receptors recycle back to the plasma membrane (A and B) or are degraded (C and D, Fig 2A) [12,23]. From the endocytic recycling compartment, receptors are recycled via path A directly to the membrane EpoR_m_ or via B to the intracellular pool (EpoR_i_). All model variants were fitted to data from our single-cell experiments.

To enrich our experimental dataset by kinetic data on EpoR synthesis and degradation, we performed two auxiliary experiments. First, Epo-GFP expressing H838 cells were bleached by applying a short laser pulse. Thereafter replenishment due to EpoR-GFP synthesis was followed in ten treated H838 cells by recording the increase of the GFP signal. Furthermore, EpoR-GFP degradation was studied in seven single H838 cells treated with cycloheximide (CHX) at a concentration of 5μg/ml to inhibit protein translation and by recording the subsequent decrease of the GFP signal. Inhibition of translation by CHX was similarly used in previous systems biological studies to quantitatively study protein degradation [50–52].

The rationale for doing these additional experiments on EpoR synthesis and degradation was that the trafficking dynamics in unperturbed experiments are likely to be a complex superposition of EpoR endocytosis, recycling, synthesis and degradation effects. Therefore, we assumed that a combination with EpoR synthesis and degradation experiments were required to make kinetic parameters for EpoR turnover identifiable. In general, combining experiments on receptor trafficking with experiments on receptor turnover is reasonable because time scales of these processes might be different.

For all model variants, cell ensemble models were constructed [40]. In the cell ensemble models, each single cell of a heterogeneous cell population was described by the same set of ODEs, and cell-to-cell variability was introduced by allowing receptor trafficking parameters and initial EpoR concentrations to be different between cells (as further described below). Cell ensemble models comprised single-cell models for Epo internalizing cells, and simplified models for photobleached and CHX treated cells, in which reactions for Epo uptake were excluded. One single-cell model describing an Epo treated cell contained 6 ODEs and between 7 and 11 parameters (S1–S3 Tables; for details, see S2 Text). Models of photobleached cells contained a reduced set of reactions describing only synthesis, degradation, transport of the EpoR between the plasma membrane and the intracellular pool, and an additional reaction describing removal of detectable EpoR species by photobleaching. Trajectories of CHX treated cells, in which synthesis was inhibited, were described by ODE models describing EpoR degradation and transport between the plasma membrane and the intracellular pool of EpoR (S4 and S5 Tables). Models of photobleached cells consisted of 3 ODEs with 5 kinetic parameters while models of CHX treated cells contained 3 ODEs with 3 kinetic parameters.

The parameters for Epo binding and unbinding, k_on,Epo_ and k_off,Epo_ were defined as being equal for each single-cell model, whereas all other kinetic parameters were allowed to vary between cells. This assumption was made, because k_on_ and k_off_ are biophysical constants, whereas receptor trafficking parameters describe lumped reactions that are controlled by concentrations of various intracellular regulatory proteins. Hence, in line with previous studies, we assumed in our model that cell-to-cell variability arises from heterogeneous expression of cellular proteins [40,53].

An ensemble model describing the complete available dataset of 16 Epo treated, 10 photobleached, and 7 CHX treated cells comprised between 156 and 220 kinetic parameters. Experimental single-cell datasets for GFP and mCherry fluorescence were linked via scaling factors to model variables in absolute concentration units. Taking together kinetic parameters, scaling factors, and initial concentrations [EpoR_m_](t_0_) and [EpoR_i_](t_0_) resulted in a total number of 230 to 294 parameters for different model variants, which were estimated by model fits of a total of 3996 data points. To estimate the scaling factor between normalized GFP fluorescence intensities in cellular compartment ROIs and absolute receptor amounts, average total cellular EpoR-GFP levels were determined by quantitative immunoblotting (S3 Fig). Immunoblotting and image stack segmentations showed that each cell contained on average 142.000 receptors and had a mean volume of about 5.47pl, which resulted in an average cellular concentration of [EpoR]_tot_ = 43.1nM.

Fitting cell ensemble models to sets of single cells treated under different conditions, i. e., by adding Epo-Cy5.5, CHX or bleaching, can in principle lead to systematic differences between sets of estimated kinetic parameters. However, this is unlikely because the same cell line was used in all conditions. Therefore, kinetic parameters of cells treated under different conditions should follow the same probability distribution [40]. Because kinetic parameters of single cells implicitly depend on concentrations of regulatory proteins that are typically log-normally distributed in cell populations [54,55], we assume log-normal distributions of single-cell parameters for EpoR trafficking processes, EpoR synthesis and degradation. To minimize differences between parameter distributions for the three experimental data sets generated by adding Epo-Cy5.5, CHX or bleaching, we added constraint terms to the likelihood function used for parameter estimations, which penalized for differences in parameter means and variances between experimental sets (for details, see S2 Text). Restricting parameter estimations by these constraint terms was advantageous with regard to model discrimination and parameter identifiability, as described below.

We found that the model variant “ACD”, with parts for direct EpoR recycling to the plasma membrane (part A) and EpoR degradation with either exocytosis (part C) or intracellular accumulation of consumed Epo (part D), could significantly better explain the set of experimental data than the other variants (Fig 2B). This was indicated by the smallest values for the corrected Akaike information criterion (AIC_corr_), which finds the most parsimonious model by weighing the number of parameters with goodness of fit and experimental noise, thereby preventing overfitting.

**Fig 3:**
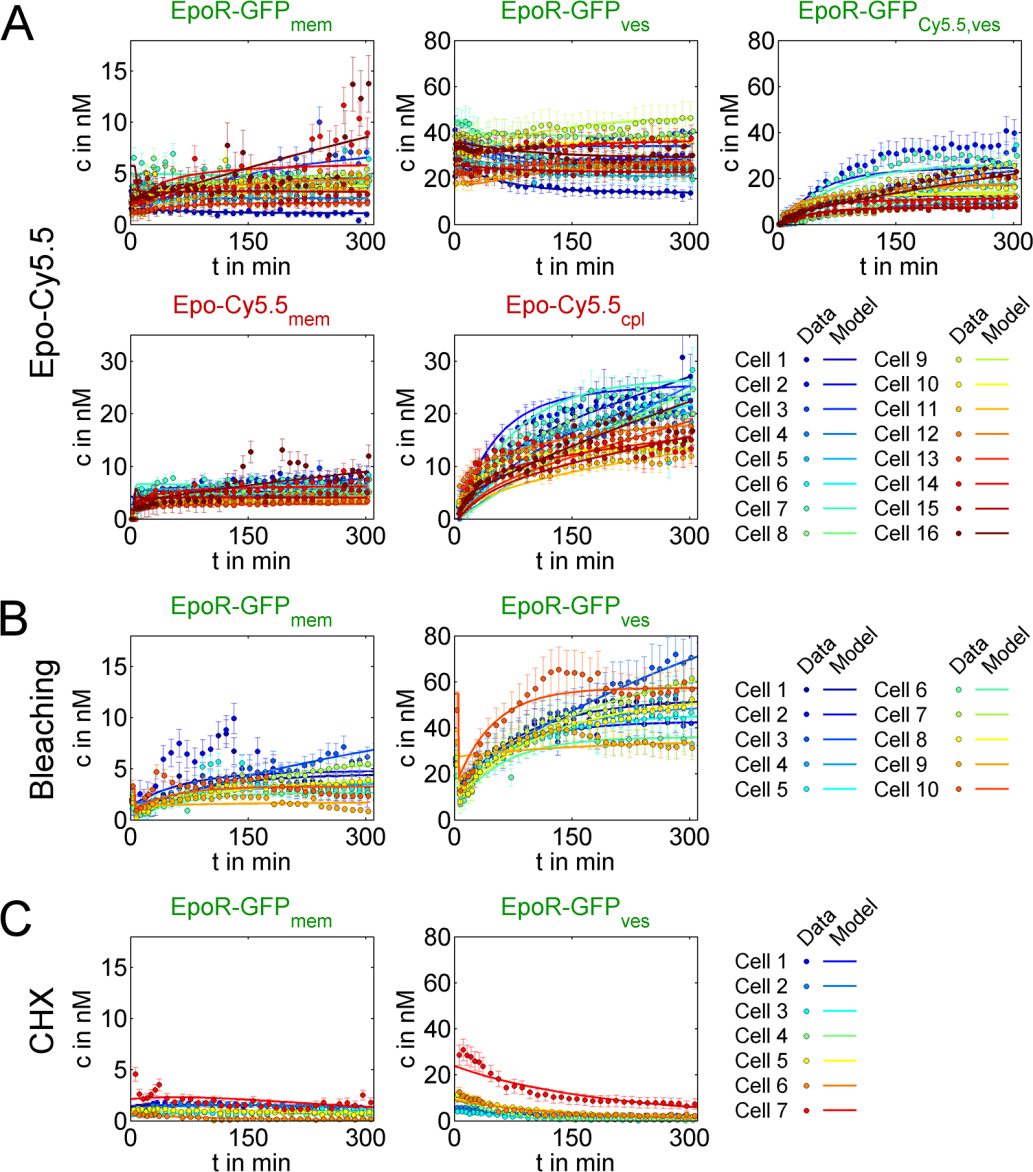
Fits of the optimal cell ensemble model variant to the complete dataset. **(A)** Fits of the model variant ACD to 16 Epo-Cy5.5 internalizing cells (circles, experimental data; lines, model fits; EpoR-GFP_mem_, membrane EpoR; EpoR-GFP_ves_, EpoR in vesicles without Epo; EpoR-GFP_Cy5.5,ves_, EpoR in Epo-Cy5.5 vesicles; Epo-Cy5.5_mem_, Epo-Cy5.5 bound to membrane EpoR; Epo-Cy5.5_cpl_, cytosolic Epo-Cy5.5). **(B)** ACD model fits to data from 10 cells newly synthesizing EpoR-GFP after bleaching at t = 5’ (EpoR-GFP_mem_, membrane EpoR; EpoR-GFP_ves_, Epo in vesicles). **(C)** ACD model fits to data from 7 cells degrading EpoR-GFP after inhibiting synthesis with CHX as in (B).

Next, we compared the model selection results for different sets of experimental data. Thereby, we assessed to which degree cell ensemble models including constraint improved the model discrimination. Already the comparison between cell ensemble models calibrated solely with data from Epo-treated cells showed that the variant “ACD” performed significantly better than the other variants. Including data for bleached and CHX treated cells further increased the AIC_corr_ difference to other variants and allowed more distinct model discrimination. In contrast, fitting model variants to data from only a single cell, instead of fitting cell ensemble models to data from several cells simultaneously, was not sufficient to determine an optimal model variant (Fig 2C), a situation comparable to conventional ODE models calibrated only with population average data, which ignore cell-to-cell variability. The optimal model variant ACD is visualized in Fig 2D. The complete set of single-cell data for Epo internalizing, bleached or CHX treated cells is shown together with the best-fit ACD model trajectories in Fig 3. In addition, scatter plots of experimental data plotted against corresponding model simulations are shown in S4 Fig, and residuals as well as residual distributions are shown in S5 Fig. Overall, it can be concluded that, our set of single cell data could be well explained by the model. The kinetic parameters associated with the reactions (grey text in Fig 2D) are further analyzed below.

**Fig 4:**
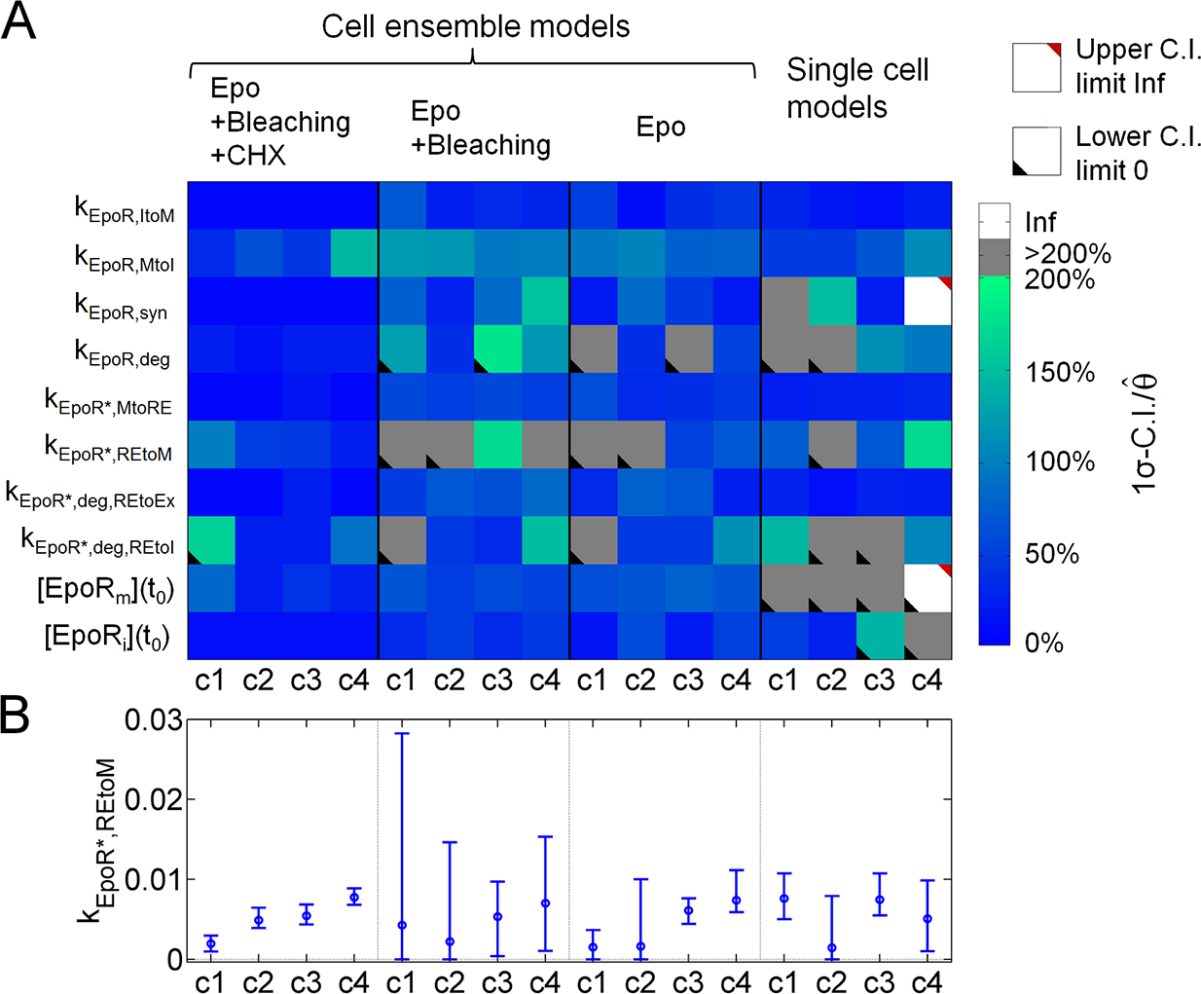
Identifiability of single-cell parameters after using different datasets for model fitting. **(A)** Relative parameter 1σ-confidence intervals (C. I.) from PLE for four exemplary cells (c1 to c4) at four different conditions for model fitting. These cells were either part of cell ensemble models (Epo internalizing + bleached + CHX treated cells, Epo internalizing + bleached cells, Epo internalizing cells only) or independent single-cell models of the variant ACD. Colors indicate the percentage of the parameter C. I. sizes relative to the value of the best fit parameters from 1000 fits for all single-cell parameters (grey color, C. I. larger than 200% of the best fit parameter; white color, C. I. of infinite size; upper or lower triangle, upper limit is infinity or lower limit is zero). While some parameters were identifiable for all four model fitting conditions, identifiability was best for including the complete dataset. **(B)** Best fit values and error bars indicating C. I. sizes from PLE for the exemplary parameter k_EpoR*,REtoM_ that describes EpoR recycling back to the plasma membrane.

We hypothesized that cell ensemble models improved parameter estimations by combining complementary experimental datasets. To test this, we analyzed parameter identifiability for different combinations of datasets in cell ensemble models in comparison to individual single-cell models. Fig 4A visualizes relative confidence interval sizes, confidence intervals divided by parameter values, obtained from profile likelihood estimation (PLE) for parameters of four exemplary cells and different experimental datasets in a color-coded manner, Fig 4B for an exemplary parameter as error bars. Essentially, confidence interval sizes decreased significantly when using cell ensemble models instead of models fitted to data from one cell at a time, and for fitting cell ensemble models to data from all three experimental conditions instead of only Epo internalizing cells. For all parameters estimated in cell ensemble models, upper confidence intervals were defined by PLE. Only for few parameters, lower confidence intervals included zero indicating that those parameters were not identifiable and that involved reactions might be eliminated in these cells. Similarly, standard deviations from the best 0.5% of 1000 fits, ordered according to their squared sum of residuals, for all model parameters showed that combining datasets for Epo-internalizing H838 cells, bleached H838 cells and CHX treated H838 cells significantly improved the accuracy of single-cell parameter estimations (S6–S8 Figs). In absence of constraint terms (S8 Fig), single-cell estimates of EpoR transport parameters were of similar magnitude as in presence of constraint terms (S6 Fig and S7 Fig) which indicates that including constraint terms improved the identifiability of single-cell parameters but did not affect the variabilities of single-cell parameters. The globally defined parameters for Epo binding and unbinding were not identifiable, which were, however, not in the focus of this study. All scaling factors were identifiable with small confidence intervals (S6 Table and S6 Fig).

In summary, we found that the EpoR model variant ACD was optimal, which is consistent with EpoR trafficking reactions described in the model by Becker et al. that was developed based on cell population average data [12]. In comparison to the model by Becker et al., our model additionally accounts for the intracellular pool of free EpoR, synthesis and degradation of the EpoR. We observed that our EpoR model could not be further reduced but that all components were required to explain the experimental data. Using cell ensemble models allowed clear discrimination between model variants and improved parameter identifiability. Improving the identifiability of single-cell parameters was necessary to analyze correlations between kinetic parameters within a population of cells, which will be further described below.

### Model analysis confirms fast EpoR transport and contribution of receptor recycling

After determining an optimal model variant, we asked how sub-compartment receptor pools remained largely unchanged in the presence of Epo and why intracellular ligand accumulation was slow. We investigated how EpoR trafficking reactions effectively contributed to these experimental observations. To this end, we extracted the concentrations of EpoR species from the model and analyzed fluxes (concentration changes per minute) through each of the reactions for each cell and at different time points.

**Fig 5:**
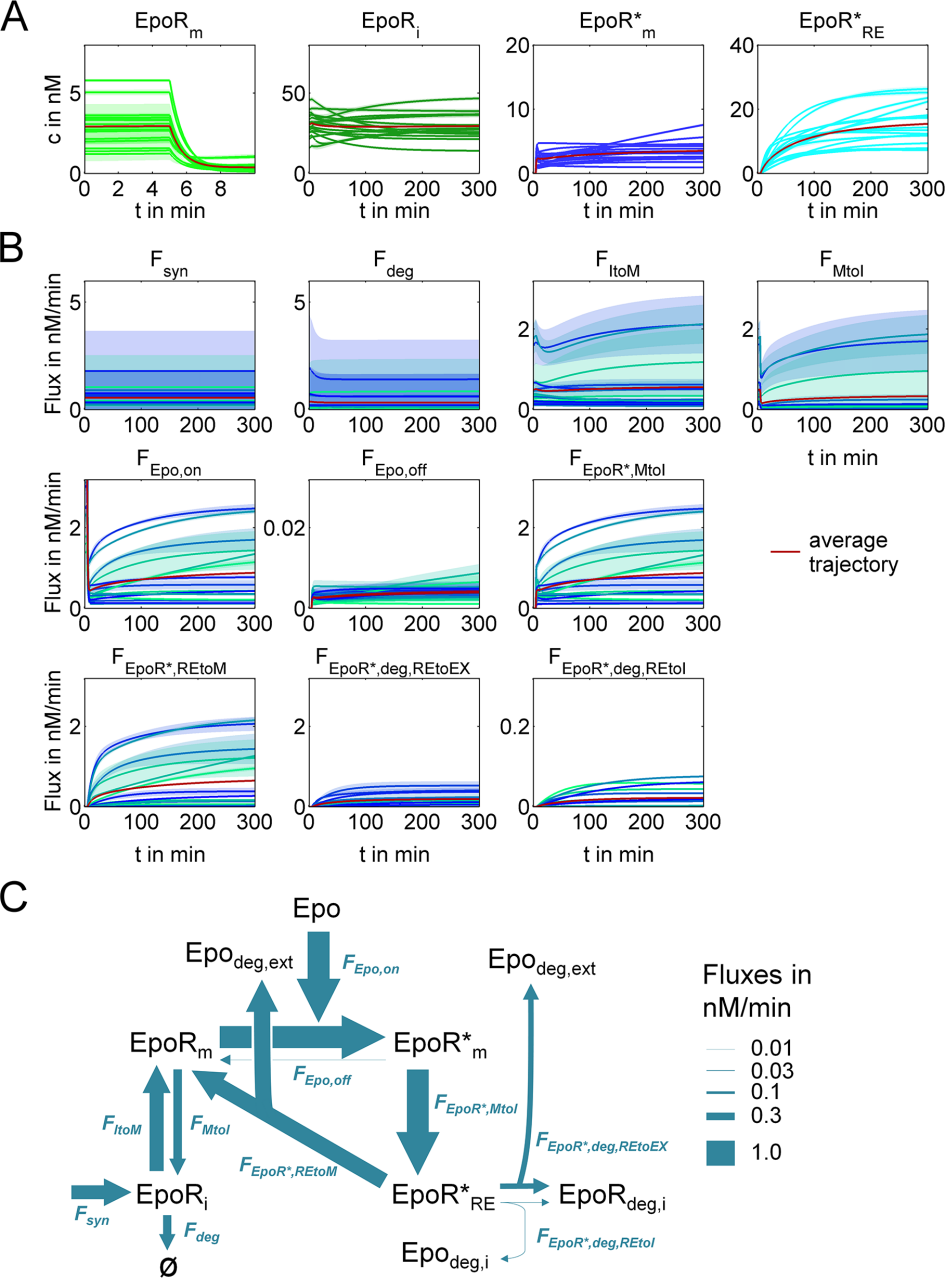
Quantitative characterization of single-cell EpoR transport dynamics. **(A)** Predicted single-cell EpoR concentration trajectories. Predictions were plotted for means of the best 0.5% of 1000 ACD model fits. Shaded areas indicate 1σ-confidence intervals. (EpoR_m_, membrane bound EpoR; EpoR_i_, intracellular EpoR; EpoR*_m_, membrane bound Epo-EpoR complexes; EpoR*_RE_, intracellular Epo-EpoR within the “recycling endosomal” compartment). Average fluxes are shown in red together with shaded areas indicating 1σ-confidence intervals, which are in most cases negligibly small. **(B)** Predicted reaction fluxes for EpoR traffic and Epo binding reactions. Lines represent means of the best 0.5% of 1000 ACD model fits, and shaded areas indicate 1σ-confidence intervals. Average fluxes are shown in red together with shaded areas indicating 1σ-confidence intervals (F_deg_, EpoR degradation; F_ItoM_ and F_MtoI_, transport from the intracellular compartment to the plasma membrane or in the opposite direction; F_Epo,on_ and F_Epo,off_, Epo binding and unbinding; F_EpoR*,MtoI_, endocytosis of Epo-EpoR; F_EpoR*,REtoM_, recycling to plasma membrane; F_EpoR*,deg,REtoEx_, degradation with exocytosis of Epo; F_EpoR*,deg,REtoI_, degradation with intracellular accumulation of degraded Epo). **(C)** Average single-cell EpoR reaction fluxes close to steady state at t = 300’, illustrated by arrow widths, show the important involvement of rapid exchange between intracellular and membrane compartments, Epo-EpoR internalization and transport back to the plasma membrane.

Model predictions of single-cell concentrations of EpoR_m_, EpoR_i_, EpoR*_m_ and EpoR*_RE_, and reaction fluxes for all EpoR reactions are shown in Fig 5A and 5B. We superposed means and standard deviations for the best 0.5% of 1000 fits for single cells and average fluxes (Fig 5B). After adding Epo, the largest fraction of the EpoR at the plasma membrane is quickly bound to Epo. The transport from the intracellular pool of EpoR (EpoR_i_) to the plasma membrane compensates for the internalization of Epo-bound EpoR (EpoR_m_*) resulting in EpoR concentrations, which are, in agreement with characteristics observed in single-cell trajectories (Fig 1), almost at steady state. Fluxes for EpoR recycling (F_EpoR*,REtoM_) reach similar magnitudes as fluxes of unoccupied EpoR from the intracellular pool to the plasma membrane (F_ItoM_). Reaction fluxes in different cells varied approximately by a factor of ten implying that EpoR transport dynamics and the consumption of Epo strongly diverge between cells, an observation, which is further analyzed below. Average fluxes at the end of the experiment (t = 300’), when fluxes were close to steady states, are illustrated in Fig 5C. Analysis of fluxes showed that a large fraction of internalized EpoR was recycled to the plasma membrane (F_EpoR*,REtoM_), while a smaller receptor fraction was degraded, mostly with exocytosis of Epo. Notably, about one percent of the total amount of free EpoR cycles per minute between the plasma membrane and the intracellular compartment (F_ItoM_, F_MtoI_).

To conclude, similar to previous studies [11,12] we observed an important contribution of receptor recycling and the fast transport of the receptor between the plasma membrane and the cytosol, and showed that the reaction fluxes varied approximately up to an order of magnitude between different cells.

### Analyzing single-cell kinetic parameters shows that correlated kinetics of opposing transport processes buffers cell-to-cell variability

Next, we addressed how the observed strong variability in reaction fluxes affects signal transduction. Specifically, we asked how the concentration of Epo-EpoR complexes at the plasma membrane indicative for the fraction of activated receptors [11,12], and the concentration of internalized Epo-EpoR complexes were dependent on EpoR transport processes.

**Fig 6:**
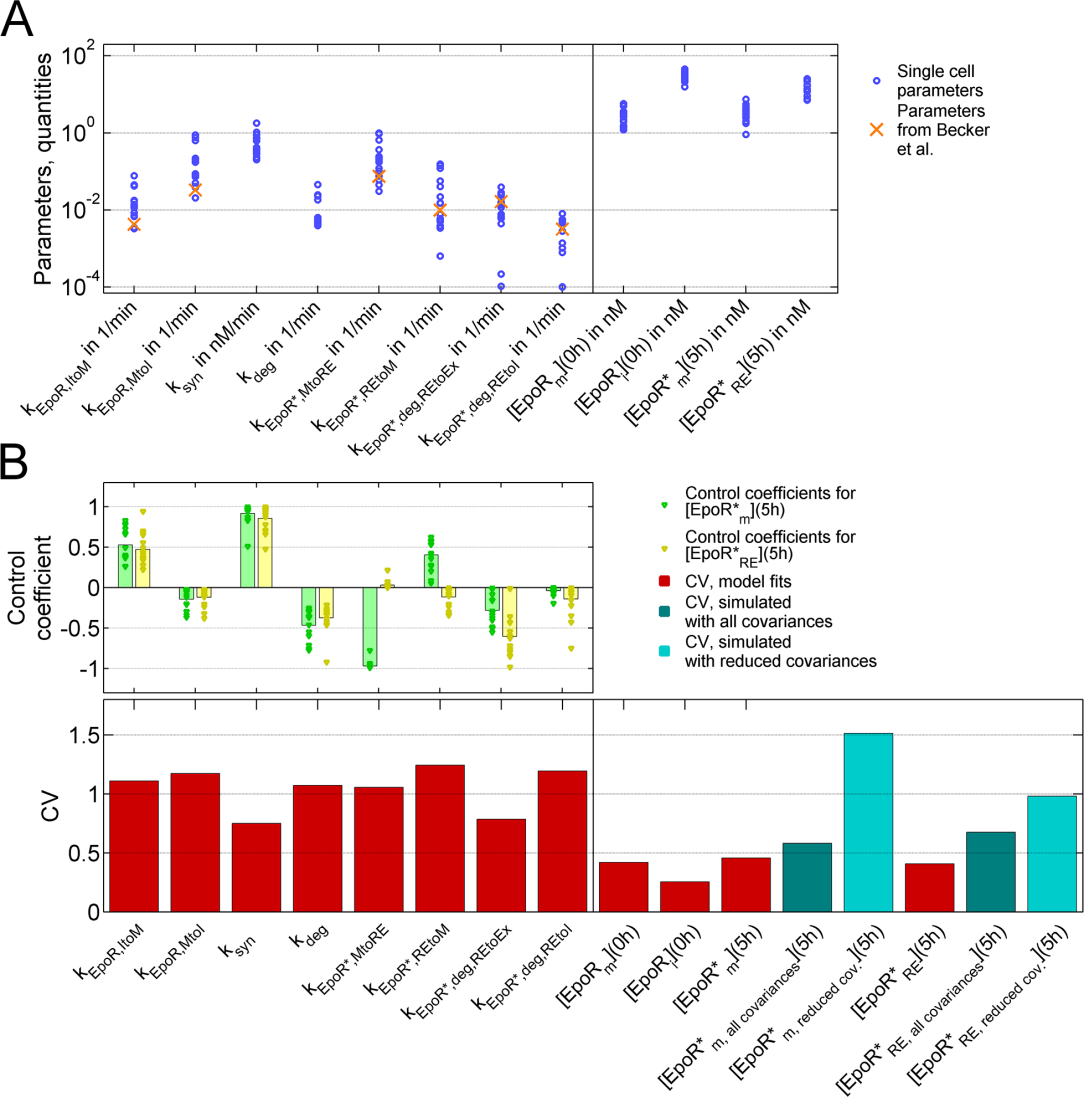
Variability of single-cell parameters. **(A)** Single-cell parameters (means of the best 0.5% of 1000 fits), as described in Fig 2D, and EpoR concentrations after fitting the ACD model to the complete dataset. Parameters available from the model by Becker et al. that was based on cell population average data are indicated. **(B)** Coefficients of variation (CV) for estimated parameters, [EpoR*_m_](5h) and [EpoR*_RE_](5h) as in (A), simulated single-cell concentrations [EpoR*_m_](5h) (bottom), and control coefficients for single-cell kinetic parameters (top). Simulated CVs were obtained by sampling 10^3^ single-cell parameter vectors from multivariate distributions, which were derived from parameter estimates after model fitting to the experimental dataset. When using all covariances between estimated parameters ([EpoR*_m, all covariances_](5h) and [EpoR*_RE, all covariances_](5h)) for sampling parameter vectors, the CVs from simulations were similar to the CVs for estimates from experimental data. Neglecting covariances for parameters describing reactions of Epo-ligated EpoR (k_EpoR*,MtoRE_, k_EpoR*,REtoM_, k_EpoR*,deg,REtoEx_, k_EpoR*,deg,REtoI_) with other parameters ([EpoR*_m, reduced cov._](5h)) resulted in substantially larger CVs.

First, we compared our single-cell parameter estimates with the corresponding kinetic parameters from the mathematical model by Becker et al. [12]. Interestingly, although Becker et al. had used a different cellular system, the murine suspension cell line BaF3 stably expressing the EpoR instead of the human adherent NSCLC cell line H838 stably expressing the EpoR-GFP, all parameters from their population average data model were inside ranges of the single-cell parameters in our model (Fig 6A), and were significantly correlated with single-cell parameter means (ρ = 0.92, p = 0.01). As observed in the study by Becker et al., the kinetic parameters for internalization of Epo-bound EpoR (k_EpoR*,MtoRE_) were in the range of the parameters for internalization of free EpoR at the plasma membrane (k_EpoR,MtoI_), indicating that ligand binding did not substantially accelerate internalization.

To further study cell-to-cell variability, we calculated the coefficients of variation (CV), which equal standard deviations divided by means, for single-cell parameters and the concentration of Epo-EpoR complexes at the cell membrane after 5 hours of Epo-stimulation, [EpoR*_m_](5h), and of internalized Epo-EpoR complexes [EpoR*_RE_](5h), when reactions were close to a steady state.

Of note, we analyzed the variability of kinetic parameters between cells, which should not be confused with analyzing parameter variances in one single-cell model to assess whether single cell parameters can be uniquely estimated. Here, identifiability of single-cell parameters and small parameter confidence intervals were prerequisites for analyzing the variabilities of parameters in a heterogeneous population of cells.

For kinetic parameters, we observed large CVs of above one besides slightly smaller CVs of about 0.7 for the parameters for EpoR synthesis (k_syn_) and for degradation of Epo-bound EpoR with exocytosis of consumed Epo (k_EpoR*,deg,REtoEx_) (Fig 6B). However, for initial concentration estimates of EpoR, and of Epo-EpoR complexes after 5 hours, CVs had substantially smaller values between 0.2 and 0.5. To analyze this divergence in variabilities, we determined concentration control coefficients for [EpoR*_m_](5h) and [EpoR*_RE_](5h). Concentration control coefficients *r* were calculated as normalized derivatives of parameters *k* as *r* = *k*/[EpoR^*^_m_](5*h*)∂[EpoR^*^_m_](5*h*)/∂*k* or *r* = *k*/[EpoR^*^_RE_](5*h*)∂[EpoR^*^_RE_](5*h*)/∂*k*, and were expected to have values above one in case of strong sensitivity towards changes of a parameter and below one in case of weak sensitivity [56,57]. All control coefficients were smaller than one, indicating robustness of the system towards parameter changes (Fig 6B).

**Fig 7:**
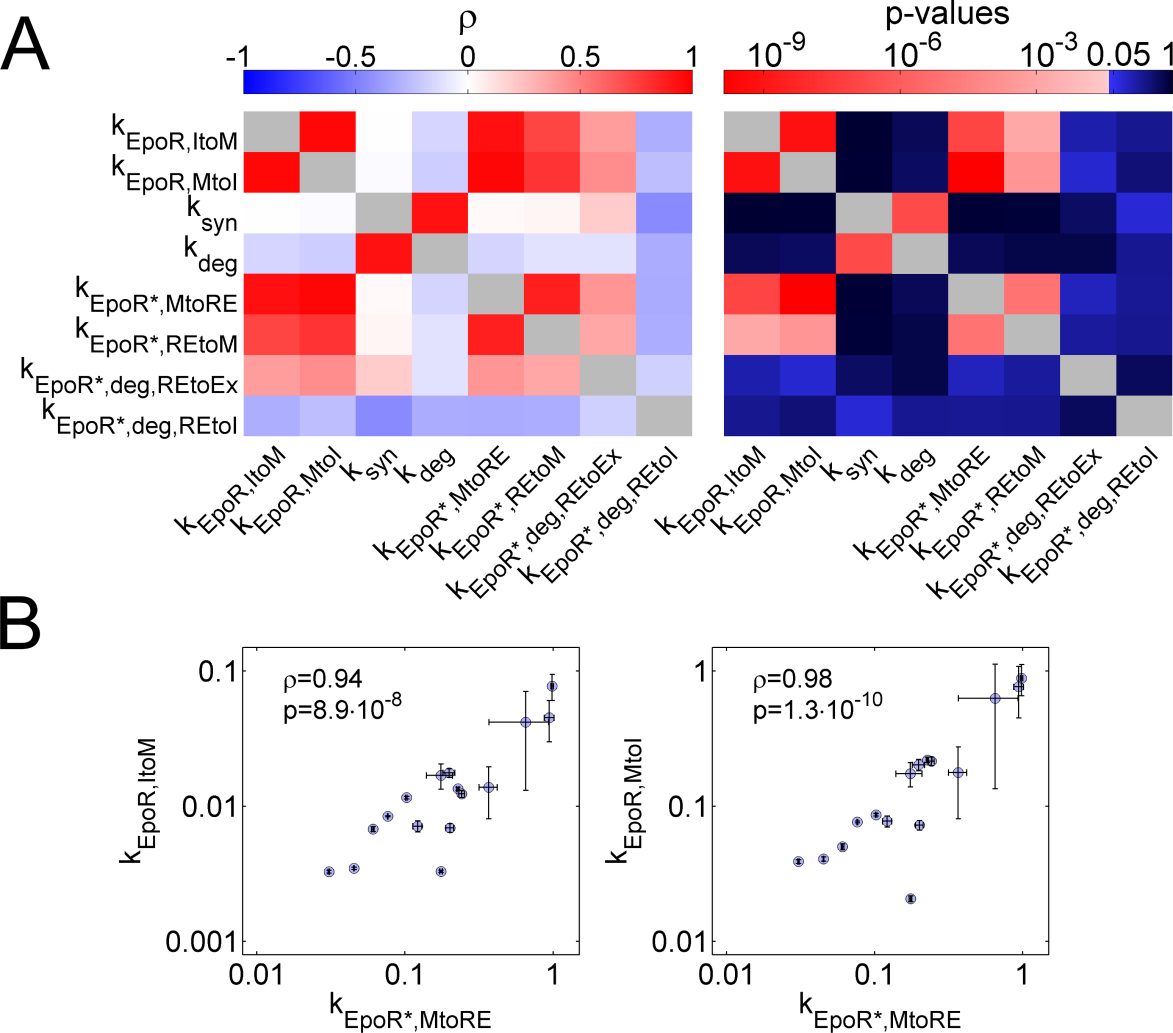
Correlations of single-cell parameters. **(A)** Correlation coefficients between single-cell kinetic parameters (left panel; white to red: positive correlation coefficients; white to blue: negative correlation coefficients) and p-values for significance of correlation coefficients obtained from t-tests (right panel; white to red: values p<0.05; blue to black: values p≥0.05). Essentially, the EpoR trafficking parameters k_EpoR,ItoM_, k_EpoR,MtoI_, k_EpoR*,REtoM_, k_EpoR*,MtoRE_ were positively correlated (ρ, Pearson correlation coefficient). **(B)** Correlations of exemplary single-cell parameters, k_EpoR,ItoM_ and k_EpoR,MtoI_, with k_EpoR*,MtoRE_. Points represent means and error bars indicate standard errors of the best 0.5% of 1000 fits.

Importantly, the strong divergence between large CVs for kinetic parameters and a small CV for the concentration of Epo-EpoR complexes after 5 hours, [EpoR*_m_](5h) and [EpoR*_RE_](5h), could be explained by positive correlations between kinetic parameters (Fig 7 and S9 Fig). In particular, the parameters k_EpoR,MtoI_, k_EpoR,ItoM_, k_EpoR*,MtoRE_, and k_EpoR*REtoM_, which described EpoR transport reactions, were positively correlated with high significance (Fig 7A and 7B, S9 Fig). The positive correlation of the parameters k_EpoR,MtoI_ and k_EpoR,ItoM_ was in line with the experimental observation that EpoR concentrations at the plasma membrane were correlated with EpoR concentrations in intracellular vesicles (Fig 1E). Further, the kinetics of processes involved in increasing and decreasing Epo-EpoR complexes at the cell membrane [EpoR*_m_] or internalized Epo-EpoR complexes [EpoR*_RE_] were positively correlated, and therefore, variabilities canceled out. Intuitively, this positive correlation between opposing processes is biochemically reasonable because different transport processes depend on the same molecular key components, such as motor proteins or constituents of the cytoskeleton [29], which will be discussed further below.

Simulating the case, in which positive correlations between kinetic parameters were removed, could further illustrate to which degree positive correlation between EpoR trafficking processes reduced noise. To this end, we derived a multivariate log-normal parameter distribution from estimates of single-cell parameters. First, we sampled vectors of single-cell parameters from the derived multivariate distribution using the complete covariance matrix, and simulated values for Epo-EpoR complexes, [EpoR*_m_] and [EpoR*_RE_], after 5 hours for each parameter vector. Then, we set covariances for parameters describing the transport of EpoR* (k_EpoR*,MtoRE_, k_EpoR*,REtoM_, k_EpoR*,deg,REtoEx_, k_EpoR*,deg,REtoI_) to zero, and again sampled parameter vectors from the modified multivariate distribution to simulate values for Epo-EpoR complexes [EpoR*_m_] and [EpoR*_RE_] after 5 hours. As expected, reducing parameter covariances resulted in a clear increase of the CV for [EpoR*_m_](5h) and [EpoR*_RE_](5h), whereas sampling from the complete covariance matrix resulted in a CV similar to the value obtained from parameter estimates after model fitting (Fig 6B). We concluded that positive correlations between single-cell parameters for intracellular EpoR transport processes reduced variability of the concentration of Epo-EpoR complexes at the plasma membrane, which implies that inter-relations between trafficking processes effectively dampened variability in the output of the system.

**Fig 8:**
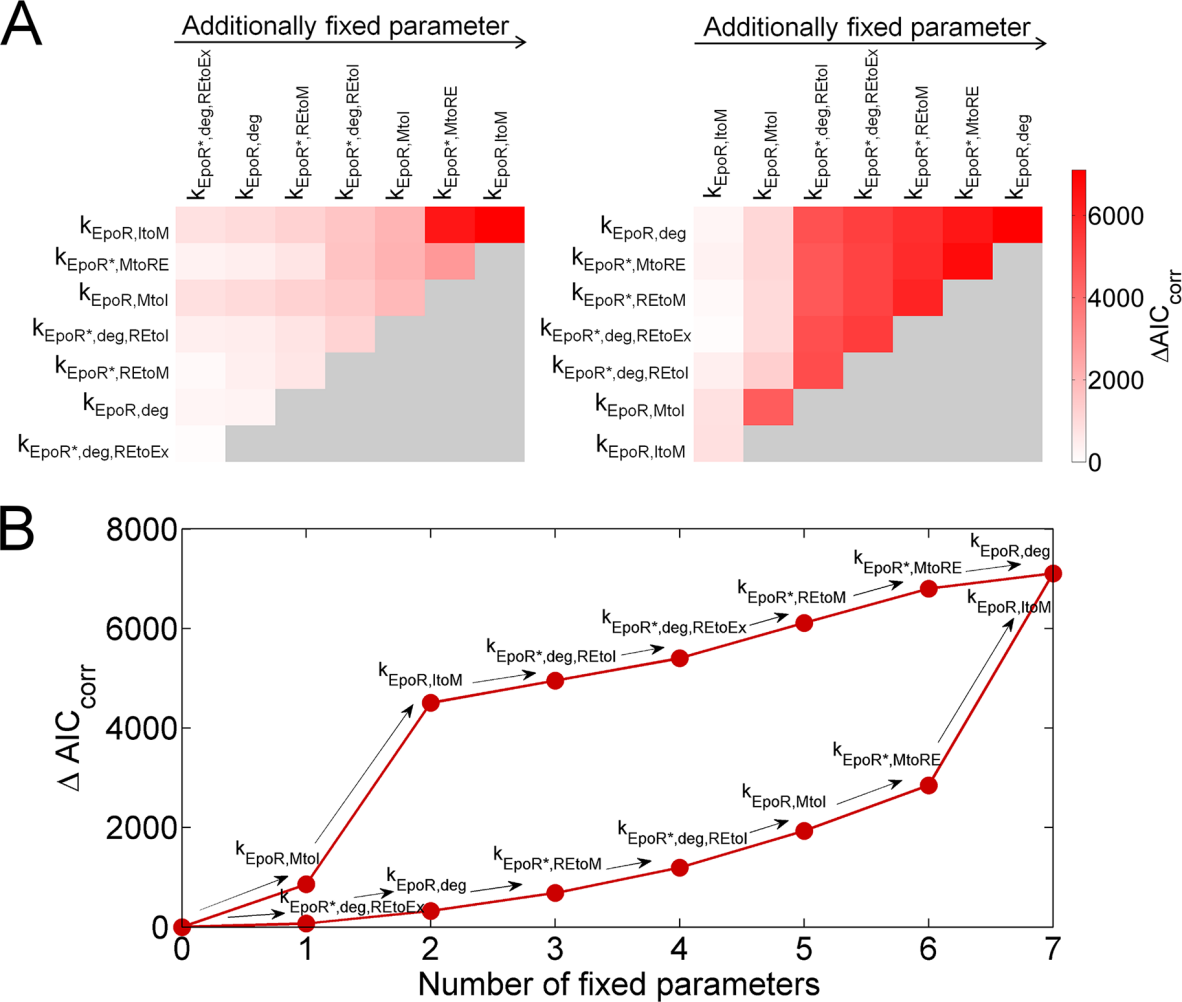
Relevance of kinetic parameter variability. **(A)** Constraining individual single-cell parameters to global parameter values that are equal for all cells lead to increases in ΔAIC_corr_ relative to the unrestricted ACD model, in which all single-cell parameters were individual. These ΔAIC_corr_ increases are shown color-coded. After fixing the single-cell parameter with the smallest (left panel) or largest increase in AIC_corr_ (right panel) to a global value, effects of fixing each of the remaining individual parameters were tested. By iterating this procedure until all parameters, except k_syn_, were fixed to global values, parameter rank orders were determined indicating to which degree the variability of different single-cell parameters contributed to explaining the experimental dataset. **(B)** Sequentially fixing single-cell parameters to global values with smallest AIC_corr_ increases (lower trajectory) or largest AIC_corr_ increases (upper trajectory) shows that variability of the parameters k_EpoR,ItoM_ and k_EpoR,MtoI_ was most important to explain cellular heterogeneity, while variability of parameters as k_EpoR,deg_ or k_EpoR*,deg,REtoEx_ was less important. The lower trajectory represents the upper row in the left graph of panel A, the upper trajectory represents the upper row in the right graph of panel A.

Next, we explored which cell-to-cell differences were essential to describe the data. We tested, whether in any of the reactions, global parameter values could be used to describe the same reactions in different cells and allow further model simplification. Single-cell parameters in the optimal model variant ACD, which were estimated individually for each cell, were sequentially defined as global parameters that were equal for all cells. Only k_syn_ was allowed to be variable in every case to account for different EpoR concentrations in individual cells. After fitting restricted model versions to the experimental dataset, differences in AIC_corr_ to the unrestricted model, in which all parameters could vary between cells, were calculated (Fig 8A and 8B). Subsequent fixing of additional parameters causing the smallest increase in AIC_corr_ showed that fixing the parameters for EpoR degradation (k_EpoR*,deg,REtoEx_, k_EpoR,deg_, k_EpoR*,deg,REtoI_) resulted only in subtle AIC_corr_ increases (Fig 8A, left panel; Fig 8B, lower trajectory) suggesting that variability of these parameters was least important. On the contrary, sequential fixing of additional parameters causing the largest increase in AIC_corr_ showed that variability of the parameters for EpoR transport to the plasma membrane (k_EpoR,ItoM_) and for EpoR internalization (k_EpoR,MtoI_) was most consequential (Fig 8A, right panel; Fig 8B, upper trajectory). Apart from the distinct impact of parameter variabilities, AIC_corr_ suggested that all variabilities were essential to fully describe the data indicating that the model could not be further simplified by assuming equal kinetic parameters for the same receptor transport processes in different cells.

To conclude, using cell ensemble models instead of separate single-cell models, and including datasets for bleached and CHX treated cells in ensemble models improved the identifiability of single-cell parameters. The dynamics of EpoR transport processes was similar in adherent H838 cells as previously described for BaF3 suspension cells. Interestingly, a positive correlation between parameter describing opposing receptor trafficking processes provides an explanation for the observed moderate cell-to-cell variability of Epo-EpoR complex concentrations at the plasma membrane despite the large variability in the kinetics of EpoR transport processes in individual cells.

## Discussion

An interesting finding of this study was that single-cell parameter estimates indicated large cell-to-cell variability in EpoR transport processes, whereas the concentration of Epo-EpoR complexes at the plasma membrane representing the activated EpoR was much less variable. Model analysis showed that the positive correlations between kinetic parameters describing opposing EpoR transport processes effectively canceled out parameter variabilities and were responsible for the dampening of cellular heterogeneity in Epo-EpoR complexes at the cell membrane and in the intracellular compartment. Receptor trafficking parameters can be assumed to result from molecular properties of receptors and on the process of vesicle trafficking. Therefore, from the perspective of cellular physiology, two explanations can be considered to explain kinetic parameter correlations. First, properties of receptor molecules, their posttranslational modifications and effects due to receptor signaling might take influence on different trafficking processes in the same manner. Second, vesicle trafficking processes that are responsible for receptor transport to the plasma membrane, internalization of ligand-bound or free receptors might be co-regulated. This appears likely because vesicle trafficking reactions share key components involved in vesicle trafficking as microtubules, myosin or actin filaments that define common paths for vesicles. In general, vesicle trafficking typically requires a small number of different motor proteins, while adaptors bound to transport protein complexes, as Rab proteins that differentially regulate transport of different cargos, are more diverse [29,58]. Transport proteins are in some aspects co-regulated [29,59], which might support synchronization of different trafficking processes. It was shown that the velocity of transport mediated by dyneins, myosins and kinesins is regulated by the concentration of ATP [60–63], and that vesicle trafficking is slowed down after loss of ATP [64,65]. Therefore, the metabolic status of the cell might determine the kinetics of different vesicle transport processes and contribute to synchronized dynamics of transport processes. Moreover, overall correlations were observed for all cellular proteins, especially for proteins involved in the same biological pathways [66,67]. For this reason, also the kinetics of more specific trafficking mechanisms might be correlated, which are dependent on classes of regulatory proteins as kinesins or Rab GTPases [68,69].

Previous studies used trafficking parameters observed at the cell population level to categorize different receptors [2,4,24,70]. Accounting for variability in receptor trafficking changes this picture because features of different functional categories of receptors might coexist in cell populations. Therefore, to fully characterize the functional properties of receptor systems in cell populations, variances and covariances of single-cell kinetic parameters have to be taken into account.

In ODE models describing comprehensively characterized cellular signal transduction networks, ODEs can reflect biochemical reactions in detail rather than summarizing several biochemical processes in single reactions. In this case, the same kinetic parameters can be assumed for different cells, and cell-to-cell variability can be inferred by different initial concentrations of involved signal transduction proteins [40,71,72]. As a consequence, correlations between signaling species become important for quantitative predictions. In a previous modeling study of programmed cell death, it was shown that for describing experimental data from a heterogeneous population of cells undergoing apoptosis, correlations between initial protein concentrations had to be taken into account to obtain realistic model predictions [53]. Furthermore, comparable to our study, correlations between initial concentrations of opposing signaling species, which were either anti- or pro-apoptotic, buffered variability of cell death times.

The motif of limiting variability by correlated kinetics of opposing reactions can be seen in the context of other mechanisms, which limit variability in biological systems such as negative feedback, for example due to ligand-dependent receptor internalization or inhibition of upstream kinases by downstream kinases, or incoherent feed-forward loops [70,73–79]. Dampening of cell-to-cell variability by co-regulation of different trafficking processes would, however, not be regarded as a direct regulatory mechanism that results from the structure of a specific signal transduction network as it is the case for negative feedback loops. In-depth analysis of how different receptor transport processes are mechanistically inter-regulated and depend on the cellular population context that was shown to be relevant for explaining cell-to-cell variability in endosomal trafficking [30], will be an important topic of future work.

In several cellular systems, single-cell dynamics significantly deviate from the behavior observed at the level of cell populations [40,42,80]. On the contrary, we observed for the EpoR that the model by Becker et al. [12], which was based on cell population average data, corresponded to the model variant that explained single-cell data best. In addition to the model by Becker et al., our model accounts for the intracellular pool of free EpoR, for EpoR synthesis and degradation. Although, that study had used a different EpoR-expressing suspension cell line, in this study, we obtained similar kinetic parameters for EpoR trafficking in adherent EpoR-GFP expressing H838 cells. This finding suggests that dynamic properties of the EpoR system are conserved between different types of cells.

We observed that internalization of Epo-EpoR complexes was not substantially accelerated compared to free EpoR, in contrast to other receptor systems, which is consistent with the finding that EpoR is internalized in a ligand-independent manner [81], and was similarly observed in BaF3 cells at the cell population level [12]. Several other receptors as the epidermal growth factor receptor (EGFR), the insulin receptor, the growth hormone receptor or the leukemia inhibitory factor receptor show substantially accelerated internalization of activated receptors [14,47,82–84], which facilitates a high temporal resolution in receptor signaling [1,3,24]. Contrarily, EpoR signaling rather depends on fast transport of EpoR between membrane and cytosolic compartments and rapid ligand depletion [11,12].

Confocal microscopy combined with 3D image segmentation was the method of choice for the time-resolved quantification of fluorescently labeled proteins in cellular compartments but offered lower throughput compared to other experimental methods for studying cellular heterogeneity as fluorescence-activated cell sorting (FACS). Nevertheless, significant correlations between single-cell parameters could be identified with the given set of single-cell data.

An essential aspect of our study was the refinement of the cell ensemble modeling approach. These advances comprised the implementation of constraint terms [40] that minimized the deviations of kinetic parameter distributions in sets of single cells treated under different experimental conditions and that were added to the log-likelihood function for parameter estimations. The approach of merging single-cell trajectories from qualitatively different experiments is widely applicable and can be transferred to various other models of cellular signaling pathways.

Taken together, we could show by combining quantitative live-cell imaging of erythropoietin receptor trafficking with mathematical modeling that receptor transport processes largely differed between individual cells. Receptor concentrations in cellular compartments were nevertheless robust to variability in trafficking processes due to the correlated kinetics of opposing transport processes.

## Materials and methods

### Cell lines and cell culture

Stable cell lines were generated from the human NSCLC cell line H838 (ATCC CRL-5844) that was purchased from American Type Culture Collection (ATCC, Manassas, VA, USA). From wild-type H838 cells, EpoR-GFP expressing cell lines were selected with 2.0 μg/ml puromycin (Sigma-Aldrich, Taufkirchen, Germany), and MyrPalm-mCherry expressing cell lines were selected with 0.8 mg/ml G418 (Sigma-Aldrich). Cell lines were maintained in DMEM (Invitrogen, Darmstadt, Germany) containing 10% fetal calf serum (Biochrom AG, Berlin, Germany), 100 μg/ml penicillin and streptomycin (Invitrogen). Medium for stably transfected cell lines additionally contained 0.2 mg/ml G418 or 0.2 μg/ml puromycin. For microscopy, cells were maintained in 8-well Lab-Tek chambers (Thermo Scientific, Asheville, NC, USA) with a density of 40.000/well. Before experiments, cells were washed and maintained in DMEM without growth factors for 3 hours to prevent basal phosphorylation of EpoR.

### Plasmids and reagents

We used the murine EpoR, which was well characterized in previous studies and is functionally equivalent to the human EpoR [12]. To express the fluorescently labeled EpoR, we utilized the retroviral expression vector pMOWS-puro encoding the murine EpoR C-terminally fused to GFP that was previously described in [85] and results in a protein of approximately 90kDa. For retroviral transduction of H838 cells, Phoenix ampho cells were transfected by the calcium phosphate precipitation method. Transducing supernatants were generated 24 hours after transfection by passing through a 0.45 μm filter (Millipore, Billerica, MA, USA). H838 cells were treated with 1ml of supernatant supplemented with supplemented with 8 μg/ml polybrene (Sigma-Aldrich) on a 6-well plate at a density of 2·10^5^ cells per well and spin-infected for 3h at 340g. Stably transduced H838 cells expressing EpoR-GFP were selected in the presence of 1.5μg/ml puromycin (Sigma-Aldrich) 24 hours after infection. The myristoylation-palmitoylation (MyrPalm) fusion construct with mCherry was a kind gift of Joel Beaudouin. It was constructed as described in [44]. To generate H838 cells stably expressing MyrPalm-mCherry and EpoR-GFP, EpoR-GFP expressing H838 cells were transfected with X-tremeGENE 9 (Roche Pharma, Basel, Switzerland) and selected with 2mg/ml G418. Cells were treated with Epo-Cy5.5 (Roche Diagnostics, Penzberg, Germany), which is a fully bioactive EpoR ligand [45]. Cy5.5 fluorescence was shown to be not pH dependent in the physiologic pH range, compared to fluorescein, because of its missing 3’-hydroxyl substituent [86].

### Analysis of total cellular lysates

Immunoblot samples were lysed with lysis buffer (20 mM Tris/HCl, pH 7.5, 150 mM NaCl, 1 mM phenylmethylsulfonyl fluoride (Sigma-Aldrich), protease inhibitor cocktail, 1% Triton X-100 (Serva, Mannheim, Germany), and 10% glycerol). Cell lysates were analyzed using SDS PAGE gels (Invitrogen). Proteins were transferred to PVDF membrane (Merck Millipore) using wet blotting. Detection was performed using the Pico Chemiluminescent Substrate from Thermo Scientific and a CCD camera (Intas, Göttingen, Germany). EpoR-GFP concentrations in EpoR-GFP-expressing H838 cells were quantified utilizing recombinant eGFP (BioVision, Mountain View, CA, USA). Cell lysates were combined with different amounts of GFP ranging from 0.2 to 10 ng and then loaded onto gels (S3 Fig). To detect EpoR-GFP and GFP in immunoblots, we used an antibody recognizing GFP (clones 7.1 and 13.1) from Roche (Basel, Switzerland). Horseradish peroxidase-conjugated secondary antibodies (Southern Biotech, Birmingham, AL, USA) were used for detection.

### Live-cell imaging

Live-cell experiments were performed in a 37°C, 5% CO_2_ incubation chamber on a CSU-22 Yokogawa spinning disk confocal (Yokogawa Electric Corporation, Tokyo, Japan) on a Nikon Ti inverted microscope equipped with 60x Plan Apo NA 1.4 objective lens (Nikon, Tokio, Japan), a Hamamatsu C9100-02 EMCCD camera (Hamamatsu Photonics, Hamamatsu, Japan) and a PerkinElmer Photokinesis bleaching/photoactivation unit (PerkinElmer, Waltham, MA, USA), using Volocity software (PerkinElmer). GFP (EpoR-GFP) fluorescence was excited at 488 nm and collected with a 527/55 emission filter (Chroma Technology Corp, Bellows Falls, VT, USA) and an exposure time of 200 ms. For bleaching, we used the FRAP module of Volocity software. Cherry (MyrPalm-mCherry) fluorescence was excited at 561 nm and collected with a 615/70 emission filter (Chroma Technology Corp) and an exposure time of 300 ms. Cy5.5 (Epo-Cy5.5) fluorescence was excited at 640 nm and collected with a 705/90 emission filter (Chroma Technology Corp) and an exposure time of 200 ms. Laser intensity was kept at a low level, at which no effect of bleaching was observed. A binning of 2x2 pixels was used. At each time point, z-stacks with 26 slides at 0.7μm step size were recorded.

In live-cell imaging experiments, cells were treated with Epo-Cy5.5 at a concentration of 4.2 nM in a total volume of 400 μl. To facilitate an even distribution of the ligand, cells were kept in 200 μl medium while recording the first image stack, before adding 200 μl Epo-Cy5.5 at a concentration of 8.4 nM to obtain the desired concentration of 4.2 nM. Within the first 30 minutes, we recorded at a time interval of 5 minutes, afterwards at a time interval of 10 minutes to obtain more densely sampled measurements at the beginning of the experiment where the signal changes were strongest.

Given an average flux F_EpoR*,ItoM_ of Epo-EpoR complexes from the plasma membrane to the cytosol of 0.8 nM/min (Fig 5B), and the average cell volume of 5.47 pl obtained from stack segmentations, the average number of Epo molecules internalized by a single cell will be about 440 molecules per minute. Therefore, within the experimental duration of 300 minutes, given the amount of 40.000 cells per well, about 3% of the total amount of Epo-Cy5.5 will be internalized in cells. It was shown that a fraction of the amount of Epo, which was secreted after internalization by cells, was still intact and could stimulate other cells [12]. Therefore, the fraction of Epo removed from the medium will be effectively less than 3%. This justifies the model assumption of constant Epo-Cy5.5 concentrations in the medium.

### Image processing

We developed custom graphical user interface-based software in MATLAB (The Mathworks, Natick, MA, USA) for segmentation of cellular compartments from image stacks (S1 Fig; for details, see S1 Text). MyrPalm-mCherry signals were used to segment the plasma membrane region of interest (ROI), EpoR-GFP and Epo-Cy5.5 signals were used to define EpoR or EpoR-Epo vesicles. To obtain observables that were proportional to variable concentrations, fluorescence intensities were normalized by cell volumes (for details, see S2 Text). Absolute volumes were calculated by multiplying voxel numbers and voxel volumes of 0.29x0.29x0.7μm^3^.

### Mathematical modeling and statistical methods

All ODE models were implemented with the MATLAB toolbox PottersWheel that was used for parameter calibrations (http://www.potterswheel.de) [87]. Model analysis and simulations were performed with custom MATLAB scripts. As a measure for the goodness of fit, we used the corrected Akaike information criterion (AIC_corr_). Model equations can be found in S1, S2, S4 and S5 Tables, and parameter estimates in S6 and S7 Tables (for details, see S2 Text). To test for linear correlation, we calculated Pearson correlation coefficients.

## Supporting information

S1 Text Image segmentation. (DOCX)

S2 Text Derivation of observables and model fitting. (DOCX)

S1 Fig Graphical user interface of the segmentation software. (DOCX)

S2 Fig Correlations between EpoR-GFP and Epo-Cy5.5 concentrations or absolute amounts. (DOCX)

S3 Fig Estimation of average EpoR-GFP amounts by calibrated immunoblotting. (DOCX)

S4 Fig Experimental measurements and corresponding model simulations. (DOCX)

S5 Fig Residuals of the best model fit of the optimal cell ensemble shown in Fig 3. (DOCX)

S6 Fig Parameter estimates from fitting variant ACD to the complete dataset. (DOCX)

S7 Fig Parameter estimates from fitting variant ACD to the data from Epo internalizing and bleached cells. (DOCX)

S8 Fig Parameter estimates from fitting variant ACD to the data from Epo internalizing cells. (DOCX)

S9 Fig Correlations between single-cell parameter estimates. (DOCX)

S1 Table Reaction rates for variants of the EpoR traffic model with variable parts A to D.
(DOCX)

S2 Table Equations of the EpoR traffic model variants. (DOCX)

S3 Table Links between observables and model variables. (DOCX)

S4 Table Reaction rates for auxiliary EpoR traffic models. (DOCX)

S5 Table Equations of the auxiliary EpoR traffic models. (DOCX)

S6 Table Global parameter and single-cell parameter estimates as shown in Fig 4. (DOCX)

S7 Table Single-cell log-normal parameter distributions. (DOCX)

S1 Movie Segmentation results for the cell shown in Fig 1A and 1B for all time points. (AVI)

S1 Dataset Single-cell data shown in Fig 3 that were used for model fitting. (XLSX)

S2 Dataset EpoR trafficking ODE model in SBML format. (XML)
