# Correction: Correlated receptor transport processes buffer single-cell heterogeneity

**DOI:** 10.1371/journal.pcbi.1006037

**Published:** 2018-03-01

**Authors:** The PLOS Computational Biology Staff

There is an update to the Funding statement. It should read: "This work was supported by the German Federal Ministry of Education and Research (BMBF, www.bmbf.de) via the MedSys-Network LungSys II (http://www.lungsys.de, FKZ 0316042C), SysTec-EpiSys (http://www.episys.org, FKZ 0315502D), the e:Bio RNA-CODE joint research project (FKZ 031A298), and an e:Bio junior group grant, the German Center for Lung Research (Deutsches Zentrum für Lungenforschung (DZL), www.dzl.de), the German Research Foundation (DFG, EXC81), and by the Networking Fund of the Helmholtz Association within the Helmholtz Alliance on Systems Biology/SBCancer (www.helmholtz.de). The authors further acknowledge financial support by the DFG within the funding programme Open Access Publishing, by the Baden-Württemberg Ministry of Science, Research and the Arts, and by Ruprecht-Karls-Universität Heidelberg. The funders had no role in study design, data collection and analysis, decision to publish, or preparation of the manuscript."
