# Supplementary material for: Correlated receptor transport processes buffer single-cell heterogeneity
Source: PLoS Comput Biol. 2017 Sep 25;13(9):e1005779. doi: 10.1371/journal.pcbi.1005779 (PMC5659801; doi:10.1371/journal.pcbi.1005779)
Supplement: S1 Text — (DOCX) [file pcbi.1005779.s001.docx]

**S1 Text. Image segmentation.**

To semi-automatically define regions of interest (ROIs) for subcellular compartments of the cells within each image stack, we developed a simple segmentation graphical user interface-based software tool (S1 Fig). After manually selecting an image region containing a single cell, ROIs were defined for the plasma membrane, the enclosed cytoplasmic compartment and vesicles therein by applying an intensity threshold value for the fluorescence signal of the membrane marker MyrPalm-mCherry and an intensity threshold value for Epo-Cy5.5 containing vesicles. A summarizing overview on the definition of ROIs and observables is given in Table A.

**Table A.** Definition of ROIs.

|  | Membrane ROI | Cytosol, outside Cy5.5 vesicles | Cytosol, inside Cy5.5 vesicles |
| --- | --- | --- | --- |
|  | Defined by outer border of the region | Defined by | Defined by intensity threshold for Cy5.5 vesicles |
| GFP intensity |  |  |  |
| Cy5.5 intensity |  |  | |

First, the intensity threshold value was determined for each cell by classifying multiple small image regions as plasma membrane or background voxels and choosing average intensity values that separate between these regions. Next, the outer border line of the area with having a line width of three pixels, which corresponds to a width of ≈1µm, was defined. This was achieved by applying a morphological erosion to define the membrane ROI, , for each two-dimensional slice of an image stack with slices. In the plane of the contact area between the plasma membrane and the specimen slide, the whole bottom area of the cell with was included in the membrane ROI.

In the volumes enclosed by the membrane ROIs, subregions containing Epo-Cy5.5 vesicles with , and residual cytosolic subregions with were selected (Table A). For this purpose, the threshold was defined. After selecting multiple small image regions classified as cytoplasmic Epo-Cy5.5 containing vesicles or cytoplasm regions outside of these vesicles, intensity values were chosen as thresholds that were used to separate between subregions. The sum of these two ROIs was equal to a ROI for the whole intracellular volume .

To visualize EpoR-GFP vesicles together with membrane ROIs (Fig 1B and S1 Movie), the threshold was defined. Again, after selecting multiple small image regions classified as cytoplasmic vesicles or cytoplasm regions outside vesicles, intensity values were chosen as thresholds that can be used to separate between subregions.
