# Supplementary material for: Correlated receptor transport processes buffer single-cell heterogeneity
Source: PLoS Comput Biol. 2017 Sep 25;13(9):e1005779. doi: 10.1371/journal.pcbi.1005779 (PMC5659801; doi:10.1371/journal.pcbi.1005779)
Supplement: S2 Text — (DOCX) [file pcbi.1005779.s002.docx]

**S2 Text. Derivation of observables and model fitting.**

**Derivation of observables**

In the model, variables for EpoR and Epo were related by scaling factors to quantities, termed observables, which were extracted from live-cell imaging data of single cells. These observables, which reflected the concentrations of EpoR-GFP or Epo-Cy5.5 in ROIs for the plasma membrane, the cytoplasm or Epo-Cy5.5 containing vesicles were defined as described in the following.

First, in each slice of an image stack at the time with for time points, regions , and were defined that contained , or voxels. In the following, voxel intensities in each slice at height with coordinates and will be denoted as . In the recording channel for GFP fluorescence, the average background intensities for each slice , which were defined by a background ROI (S1 Fig), were subtracted from sums of intensities in slides of cellular ROIs before summing up over all slides, and dividing by the total number of voxels of a cell

.

This equals summing up offset-corrected intensities and dividing by . The observable for EpoR-GFP in the for the plasma membrane ROI therefore reads

(1)

Accordingly, the observable for EpoR-GFP in the Epo-Cy5.5 vesicles ROI reads

, (2)

and the observable for EpoR-GFP in the in the residual cytosolic volume ROI

. (3)

For photobleached and cycloheximide (CHX) treated cells, an observable for membrane bound EpoR-GFP was defined according to Eq. 1. Because Epo-Cy5.5 was absent, an observable for the complete cytosolic amount of EpoR-GFP was defined by background corrected intensities in the whole intracellular regions

. (4)

For EpoR-GFP, it could be assumed that the fluorescence intensity in the channel for GFP reflected the amount of functional molecules. Because Epo was tagged with the dye Cy5.5, which was not degradable as GFP, the intensity in the Cy5.5 channel reflected not only the amount of Epo-Cy5.5 but also the amount of degraded Epo. To not be biased by possibly varying ratios between Epo-Cy5.5 and degraded Epo in different cellular compartments, we defined only one observable for the total amount of internalized Epo-Cy5.5, which represented the sum of internalized Epo-Cy5.5 and the Cy5.5 signal reflecting the amount of degraded Epo.

Estimating the background intensity for Cy5.5 fluorescence was more complicated than for GFP fluorescence because, after adding Epo-Cy5.5, the extracellular signal exceeded the intracellular fluorescence intensity. For this reason, the intracellular Cy5.5 intensity in the first time point directly after adding Epo-Cy5.5, before Epo-Cy5.5 was internalized, was taken as an offset. Accordingly, the equation for the observable for internalized Epo-Cy5.5 therefore reads

. (5)

To obtain an observable for membrane bound Epo-Cy5.5, an additional offset had to be taken into account because the segmented ROI was located in the transition between extracellular space containing Epo-Cy5.5 and the plasma membrane. As a result, the offset in the membrane ROI was slightly larger than for the intracellular ROI. After subtracting the same offset as for the observable for intracellular Epo-Cy5.5, this additional threshold was estimated together with kinetic parameters for receptor traffic by model fitting. Therefore, the equation for the cytosolic EpoR-GFP observable reads

(6)

Because it was not possible to record experimental replicates in individual cells, observable errors had to be estimated. For this purpose, smoothing splines were fit to the extracted observable trajectories [40]. Errors , , , , and were estimated as the maximum between linear error models and the standard deviation of the differences between the smoothing spline the experimental data. These linear error models were defined for observables by fitting

(7)

to the difference between the smoothing spline and the experimental data.

The resulting set of single-cell observables comprised time-resolved measurements in 16 Epo treated cells (Eqs. 1-3, 5 and 6). In 11 photobleached and 7 cycloheximide treated cells, only EpoR-GFP observables were recorded (Eqs. 1 and 4).

**Combining data from different experimental setups in a cell ensemble model**

Cellular ensemble models contain sets of ODEs, variables and kinetic parameters for each cell of an ensemble of cells. In this study, we recorded datasets for Epo-Cy5.5 internalizing, bleached and CHX treated cells and therefore obtained three sets of single-cell data. When fitting cell ensemble models to data from more than one set of single-cell data, we included additional terms in the log-likelihood sum for parameter estimations to minimize differences of kinetic parameter means and coefficients of variation (CV) between the three sets. These terms increased with differences between means and CVs for parameters of the sets of single-cell data. This procedure was justified by the fact that the same cell line was used in all three conditions. We formulated the adjusting terms in analogy to the procedure of log-likelihood based parameter estimations assuming normally distributed errors of data points. Similar as for the typical implementation of constraints in the PottersWheel toolbox, which we used for parameter estimations, we implemented weighting factors to increase the weights of the adjusting terms [86] (http://www.potterswheel.de/Pages/API/pwAddCS.html).

In general, experimental data described by a cell ensemble model can comprise experimental sets , which contain cells. These cells were described by ODE models with single-cell parameters with . Set average parameters were defined by , overall average parameters by . To minimize differences between parameter means, we used the term

. (7)

In Eq. 7, denotes a weighting factor, which can be used to account for the numbers of cells in each set . The parameter-specific weights were obtained by error propagation for the differences between and . Using standard error of the mean estimates for the average parameters and resulted in

. (8)

In a similar manner, we implemented a term to minimize differences between CVs for single-cell parameters from different experimental sets. Assuming log-normal distributions for kinetic parameters, we first transformed parameters to . Then, we applied the formula for an unbiased estimate of the CV for a small to medium sized samples of a normally distributed variable

,

in which denotes the standard deviation, and denotes the mean of . To minimize differences between CVs for each set , we calculated relative deviations between the overall average CV , and the CV for each set . To estimate errors of CVs , we applied the simplified formula for the standard error . Because our number of experimental sets was small, we estimated the error of the average CV by . Thereby, we obtained the term

. (9)

Within Eq. 9, denotes a weighting factor and a parameter specific weight, which was obtained by error propagation for the relative deviations from average CVs

. (10)

Inserting Eq. 10 with approximated errors and in the arguments of Eq. 9 results in the expression

.

Because our auxiliary models for bleached or CHX treated cells contained subsets of the single-cell parameters of the models for Epo internalizing cells, and contained contributions for parameters that appeared either under all three conditions or in the models for Epo internalizing and bleached cells (S1–S5 Tables for model definitions). and contained contributions for the parameters , and for the sets of Epo internalizing, bleached and CHX treated cells, and additionally for the parameter for the sets of Epo internalizing and bleached cells.

Furthermore, we used experimentally quantified EpoR-GFP concentrations to restrict parameter estimations. By calibrated immunoblotting, we estimated that our EpoR-GFP expressing H838 cell had on average 142.000 EpoR-GFP per cell (S3 Fig). From image segmentations, we determined an average cell volume of resulting in an average total concentration of . In each single-cell model that was part of the cell ensemble model, initial concentrations for membrane bound or intracellular EpoR, and , were individually estimated resulting in a total single-cell concentration . We used another term for minimizing relative deviations between the averages of estimated EpoR concentrations in each set, and the experimentally determined average receptor concentration. Accordingly, the term was defined by

. (11)

As in Eqs. 7 and 9, denotes a weighting factor.

It is reasonable to assume that, in the absence of Epo, EpoR transport between membrane and cytosolic compartments was at steady state. Based on this assumption, we defined another constraint term for parameter estimations to assure steady states at the start of each experiment. The difference of fluxes

for every cell in experimental set , which equals zero at steady state, was included in the constraint term. Therefore, the constraint term reads

(12)

with the weighting factor .

In summary, we defined several constraint terms, which were added to the log-likelihood function for parameter estimations, to adapt the model fitting to physiological boundary conditions. We defined the constraint terms and to minimize differences between means and variances of kinetic parameters in sets of experimental single-cell data (Eqs. 7 and 9), to minimize differences between average parameter estimates of total receptor concentrations and the experimentally determined average receptor concentration (Eq. 11), and to enforce initial steady states for EpoR transport (Eq. 12). Weighting factors , and in to were set to the numbers of cells in each set. Thereby, contributions of each experimental set in constraint terms were adjusted to set sizes. Because contained equal contributions from each cell, it was reasonable to choose a weighting factor , which was independent of set sizes. To strongly penalize for deviations of EpoR fluxes from initial steady states, we chose a value of according to the suggested procedure in the PottersWheel toolbox for defining constraints [87] (http://www.potterswheel.de/ Pages/API/pwAddCS.html).

**Fitting procedure**

Multi-start local optimizations were conducted using the MATLAB toolbox PottersWheel [87]. The table below indicates initial guesses for the first fits of fit sequences and allowed parameter ranges. In most parameters, variation over at least 4 orders of magnitude was allowed.

Initial concentrations [EpoR_m_](t_0_) and [EpoR_m_](t_0_), were restricted to an interval between 10% and 90% of the experimentally determined average EpoR concentration of 43.1 nM. It could be anticipated that these initial EpoR concentrations were identifiable because of the known average EpoR concentration in combination with experimental single-cell measurements of GFP fluorescence in compartments of the plasma membrane and the cytosol. Accordingly, S6 Fig to S8 Fig indicate that estimates of [EpoR_m_](t_0_) and [EpoR_m_](t_0_) were identifiable within the allowed intervals.

Furthermore, restrictions were made for the globally defined binding and unbinding parameters k_on,Epo_ and k_off,Epo_. These parameters were anyway unidentifiable in our experimental setup, and not in the focus of this study. For these, we used initial guesses of 0.1/(nM⸱min) and 0.01/min that were close to the previously determined values of 0.105/(nM⸱min) and 0.0172/min [12], equivalent to a K_D_ value of 0.16 nM. Estimates of these parameters were allowed to vary within the intervals [0.05/(nM⸱min); 0.3/(nM⸱min)] and [10^-3^/min; 0.05/min], respectively, to allow K_D_ values between 3.3 pM and 1 nM.

According to its definition, for the background intensity parameter I_Cy5.5,mem,th_, an upper limit of 4.2nM was set (see section “Derivation of observables” for details).

For starting the subsequent fits of a multi-start local optimization, initial parameter values were chosen in a randomized manner to assure that fits do not start from the same initial set of parameters. Parameters were fitted on the log-scale. We allowed a maximum for 600 iterations for each fit. In most cases, however, fits converged after less than 100 iterations.

**S2 Text Table.** Initial parameter values for the first fit of a fit sequence and allowed parameter intervals.

| Parameter | Initial value | Lower bound | Upper bound |
| --- | --- | --- | --- |
| Single-cell parameters |  |  |  |
| k_EpoR,ItoM_ in 1/min | 0.1 | 10^-4^ | 1 |
| k_EpoR,MtoI_ in 1/min | 0.1 | 10^-4^ | 1 |
| k_EpoR,syn_ in nM/min | 0.1 | 10^-3^ | 10 |
| k_EpoR,deg_ in 1/min | 0.1 | 10^-4^ | 1 |
| k_EpoR*,MtoRE_ in 1/min | 0.01 | 10^-4^ | 1 |
| k_EpoR*,REtoM_ in 1/min | 0.01 | 10^-4^ | 1 |
| k_EpoR*,REtoI_ in 1/min | 0.01 | 10^-4^ | 1 |
| k_EpoR*,deg,REtoEx_ in 1/min | 0.01 | 10^-4^ | 1 |
| k_EpoR*,deg,REtoI_ in 1/min | 0.01 | 10^-4^ | 1 |
| I_Cy5.5,mem,th_ in nM | 0.1 | 10^-4^ | 4.2 |
| [EpoR_m_](t_0_) in nM | 4.31 | 0.431 | 43.1 |
| [EpoR_i_](t_0_) in nM | 38.79 | 3.879 | 387.9 |
| Global parameters |  |  |  |
| k_on,Epo_ in 1/(nM⸱min) | 0.1 | 0.05 | 0.3 |
| k_off,Epo_ in 1/min | 0.01 | 10^-3^ | 0.05 |
| s_GFP,1_ in 1/nM | 1 | 10^-4^ | 10^3^ |
| s_Cy5.5,1_ in 1/nM | 1 | 10^-4^ | 10^3^ |
| s_GFP,2_ in 1/nM | 1 | 10^-4^ | 10^3^ |
| s_GFP,3_ in 1/nM | 1 | 10^-4^ | 10^3^ |
| s_GFP,4_ in 1/nM | 1 | 10^-4^ | 10^3^ |
| s_GFP,5_ in 1/nM | 1 | 10^-4^ | 10^3^ |
