# Supplementary material for: Correlated receptor transport processes buffer single-cell heterogeneity
Source: PLoS Comput Biol. 2017 Sep 25;13(9):e1005779. doi: 10.1371/journal.pcbi.1005779 (PMC5659801; doi:10.1371/journal.pcbi.1005779)
Supplement: S1 Fig — (DOCX) [file pcbi.1005779.s003.docx]

**
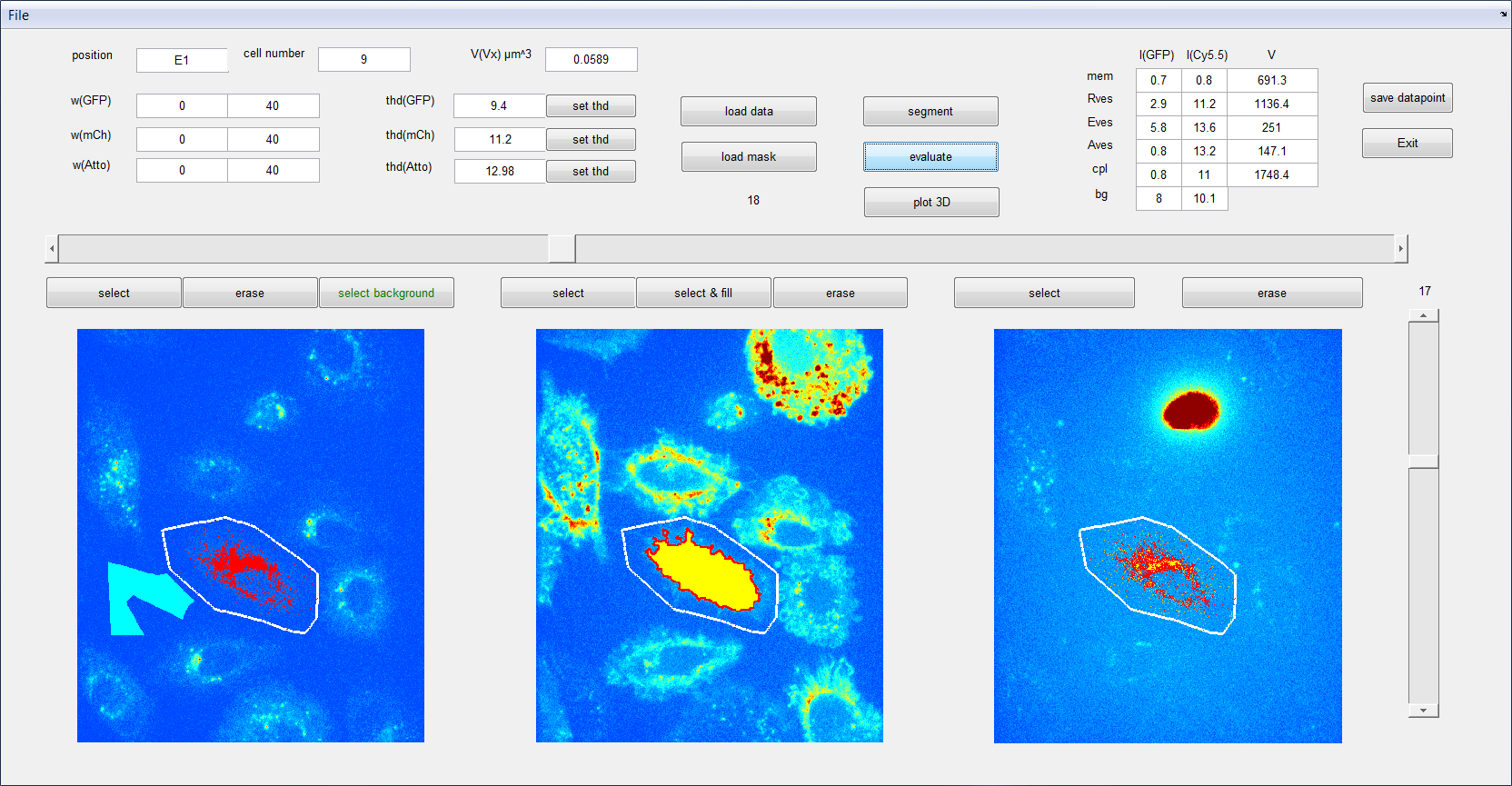
S1 Fig.** **Graphical user interface of the segmentation software.** Three windows display EpoR-GFP (left), MyrPalm-mCherry (center) and Epo-Cy5.5 (right) images and segmented ROIs. Inside predefined areas for single cells, membrane and vesicle ROIs (containing EpoR-GFP, Epo-Cy5.5 or EpoR-GFP and Epo-Cy5.5) were segmented according to intensity threshold values to determine ROI volumes and mean EpoR-GFP and Epo-Cy5.5 intensities for each time point. ROI borders can be manually corrected. Background ROIs can be manually selected to determine background GFP and Cy5.5 intensity values that were subtracted from intensities in cellular ROIs (light blue area in left image window).
