# Supplementary material for: Correlated receptor transport processes buffer single-cell heterogeneity
Source: PLoS Comput Biol. 2017 Sep 25;13(9):e1005779. doi: 10.1371/journal.pcbi.1005779 (PMC5659801; doi:10.1371/journal.pcbi.1005779)
Supplement: S2 Fig — (DOCX) [file pcbi.1005779.s004.docx]

**
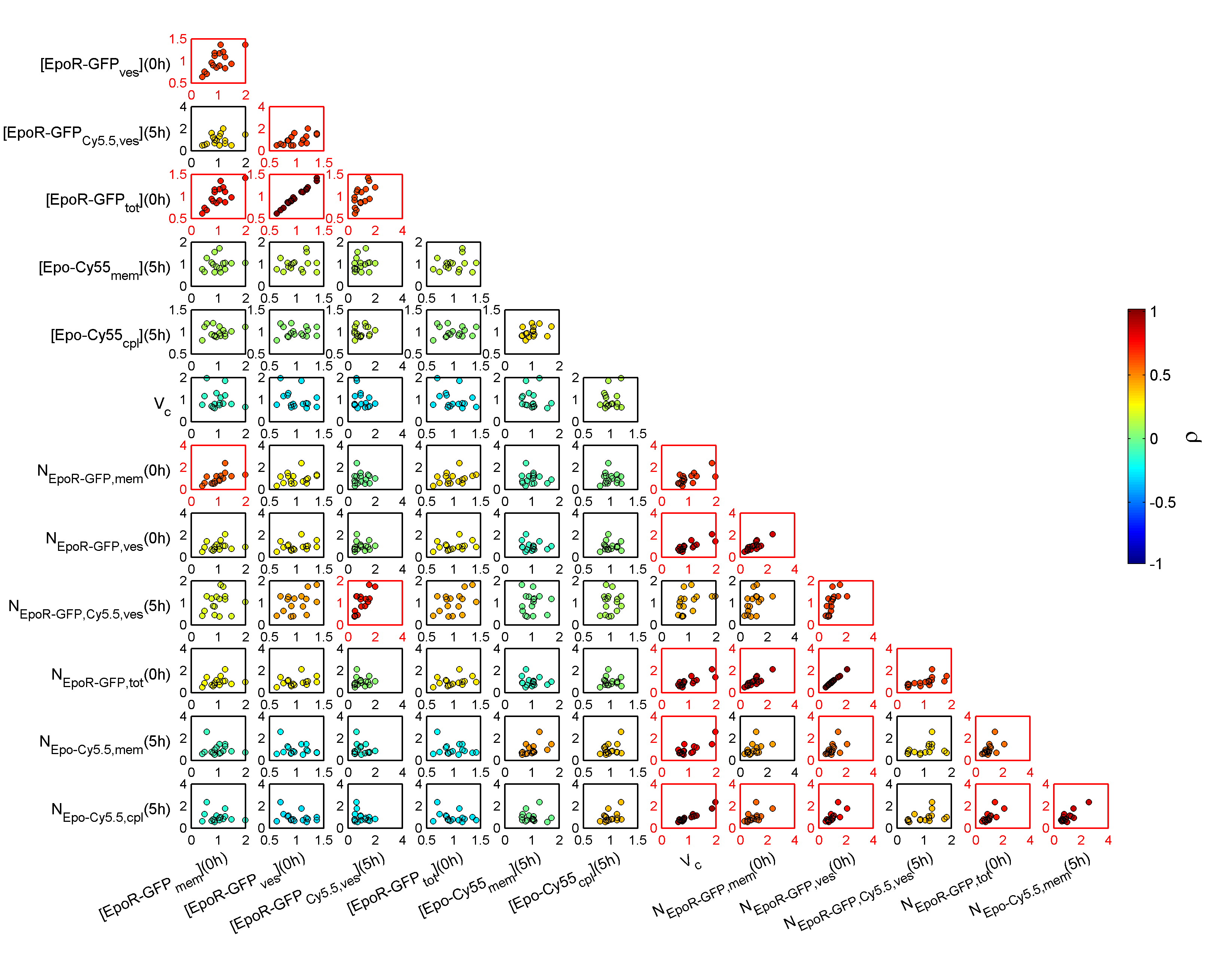
**

**S2 Fig.** **Correlations between EpoR-GFP and Epo-Cy5.5 concentrations or absolute amounts.** Correlations between fold changes of EpoR-GFP and Epo-Cy5.5 concentrations or absolute amounts for single cells relative to population averages are indicated as in Fig 1E–1G. Absolute amounts were estimated by sums of fluorescence signals in cellular compartment ROIs. Concentrations were estimated by dividing sums of fluorescence signals by cell volumes. Circle colors indicate the values of correlation coefficients and red boxes indicate significant correlation with p<0.05 (ρ, Pearson correlation coefficient). Notably, cell volumes V_c_ were in most cases significantly correlated with absolute amounts but not with concentrations. This indicates that, for describing variability in the cell population, concentrations and cell volumes might be regarded as independent random variables while absolute amounts, which equal the product of volumes and concentrations, might be regarded as dependent variables. Therefore, it is reasonable that correlations between absolute amounts were overall stronger than between concentrations. As a next step, mathematical modeling was applied to further analyze cell-to-cell variability ([EpoR-GFP_mem_], [EpoR-GFP_ves_], [EpoR-GFP_Cy5.5,ves_], EpoR-GFP concentrations in plasma membrane, EpoR-GFP or EpoR-GFP&Epo-Cy5.5 ROIs; [EpoR-GFP_tot_], total cellular EpoR-GFP concentration; [Epo-Cy5.5_mem_], Epo-Cy5.5 concentration in the membrane ROI; [Epo-Cy5.5_cpl_], cytosolic Epo-Cy5.5 concentration; N_EpoR-GFP,mem_, N_EpoR-GFP,ves_, N_EpoR-GFP,Cy5.5,ves_, absolute amounts of EpoR-GFP in membrane, EpoR-GFP or EpoR-GFP&Epo-Cy5.5 ROIs; N_EpoR-GFP,tot_, total amount of EpoR-GFP; N_Epo-Cy5.5,mem_, amount of Epo-Cy5.5 in the plasma membrane ROI; N_Epo-Cy5.5,cpl_, amount of cytosolic Epo-Cy5.5).
