# Supplementary material for: Correlated receptor transport processes buffer single-cell heterogeneity
Source: PLoS Comput Biol. 2017 Sep 25;13(9):e1005779. doi: 10.1371/journal.pcbi.1005779 (PMC5659801; doi:10.1371/journal.pcbi.1005779)
Supplement: S3 Fig — (DOCX) [file pcbi.1005779.s005.docx]

**
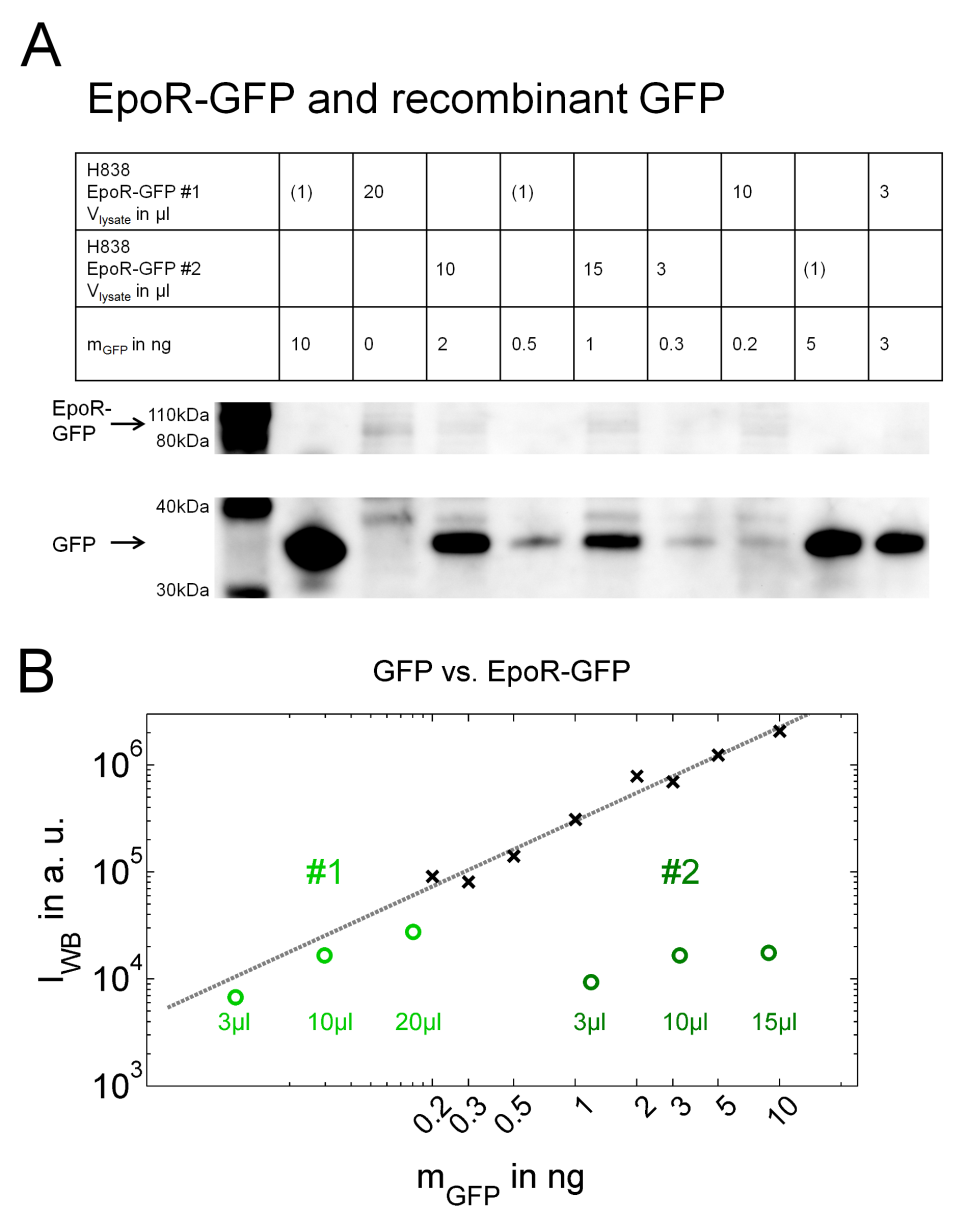
**

**S3 Fig.** **Estimation of average EpoR-GFP amounts by calibrated immunoblotting. (A)** Lysates of two EpoR-GFP expressing H838 cell clones, #1 and #2, were mixed with doses of recombinant GFP, blotted, and detected with anti-GFP antibody. Values in brackets indicate bands with low signals close to background intensity that were excluded from evaluations. **(B)** Calibration curve of GFP (crosses) and amounts of EpoR-GFP in different volumes of lysate (circles). Intensities were background corrected. A part of the blot between 40kDa and 80kDa was cut out. Dividing the rescaled intensities by the numbers of cells in lysates (and) resulted in estimates of N_EpoR-GFP_=1.45·10^5^ (SEM = 0.12·10^5^) EpoR-GFP per cell for clone #1 and N_EpoR-GFP_=1.42·10^5^ (SEM = 0.48·10^5^) for clone #2. In all further experiments, clone #2 was used.
