# Supplementary material for: Correlated receptor transport processes buffer single-cell heterogeneity
Source: PLoS Comput Biol. 2017 Sep 25;13(9):e1005779. doi: 10.1371/journal.pcbi.1005779 (PMC5659801; doi:10.1371/journal.pcbi.1005779)
Supplement: S4 Fig — (DOCX) [file pcbi.1005779.s006.docx]

**
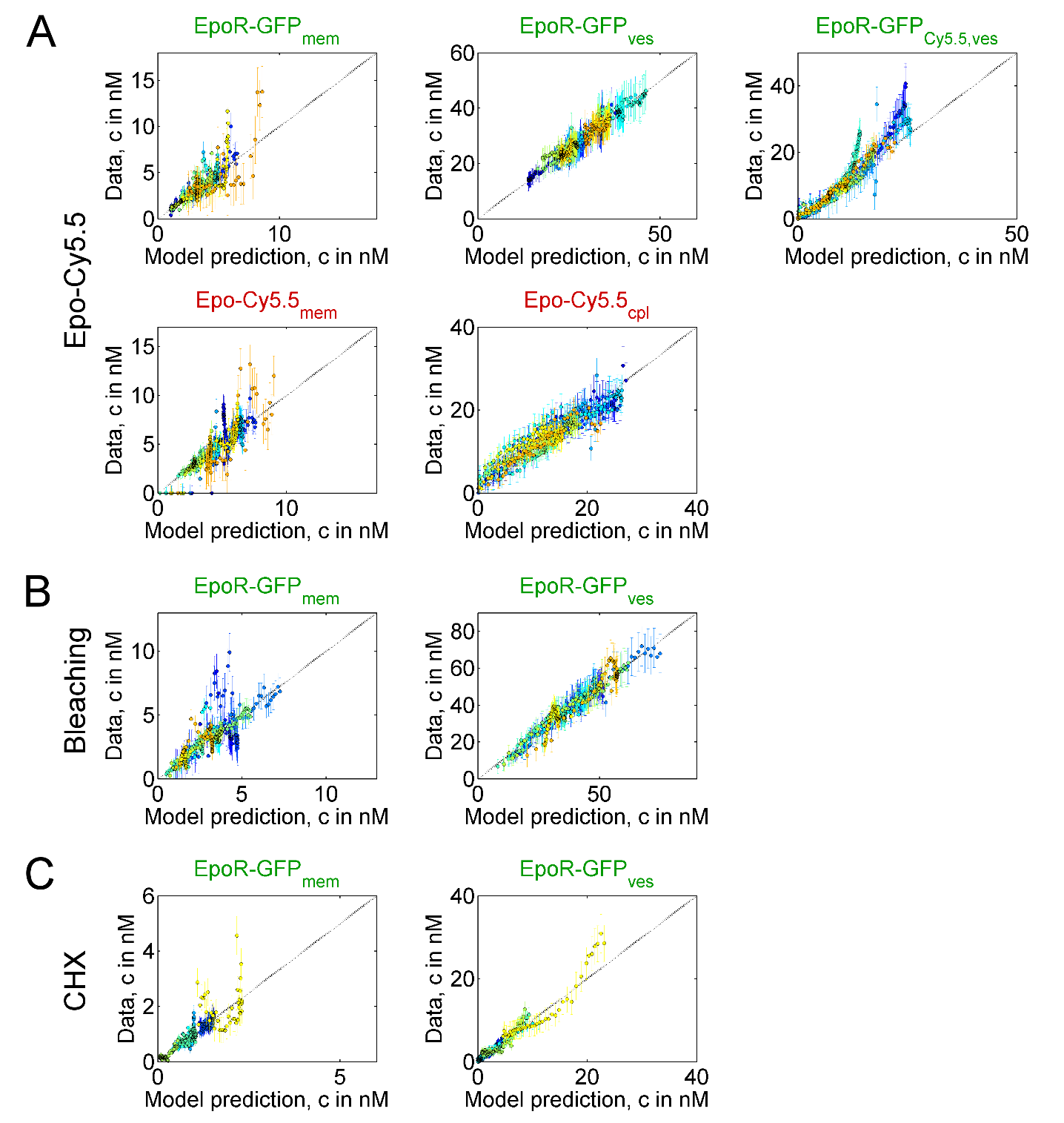
**

**S4 Fig.** **Experimental measurements and corresponding model simulations.** **(A)** Data points from Epo-Cy5.5-treated cells were plotted against model simulations of the best fit of the optimal model variant ACD to the complete dataset of Epo-internalizing, bleached or CHX-treated cells. Notably, most points are located on the diagonal (EpoR-GFP_mem_, membrane EpoR; EpoR-GFP_ves_, EpoR in vesicles without Epo; EpoR-GFP_Cy5.5,ves_, EpoR in Epo-Cy5.5 vesicles; Epo-Cy5.5_mem_, Epo-Cy5.5 bound to membrane EpoR; Epo-Cy5.5_cpl_, cytosolic Epo-Cy5.5). **(B)** Data points from bleached cells as in (A) (EpoR-GFP_mem_, membrane EpoR; EpoR-GFP_ves_, Epo in vesicles). **(C)** Data points from CHX-treated cells as in (A).
