# Supplementary material for: Correlated receptor transport processes buffer single-cell heterogeneity
Source: PLoS Comput Biol. 2017 Sep 25;13(9):e1005779. doi: 10.1371/journal.pcbi.1005779 (PMC5659801; doi:10.1371/journal.pcbi.1005779)
Supplement: S5 Fig — (DOCX) [file pcbi.1005779.s007.docx]

**
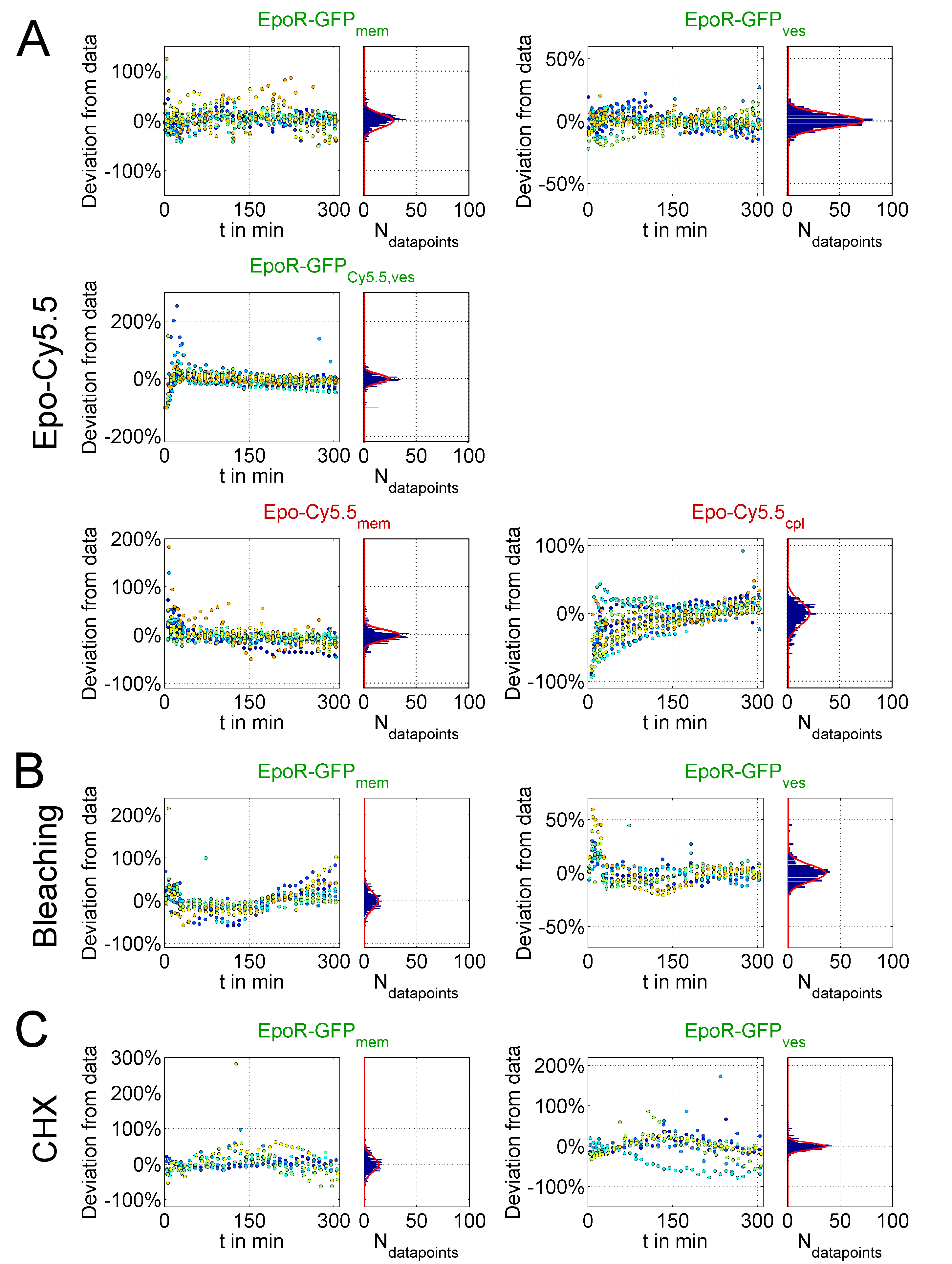
**

**S5 Fig.** **Residuals of the best model fit of the optimal cell ensemble shown in Fig. 3.** **(A)** Residuals for Epo-Cy5.5-treated cells are shown as relative differences between model fits $y_{model}$ and experimental data $y_{experiment}$, divided by experimental data $\left(y_{model}-y_{experiment}\right)/y_{experiment}\cdot100\%$. Left panels show residuals for timepoints, and right panels show histograms with gaussian fits (red lines) centered at zero. Gaussian fits indicate that residuals are about normally distributed (EpoR-GFP_mem_, membrane EpoR; EpoR-GFP_ves_, EpoR in vesicles without Epo; EpoR-GFP_Cy5.5,ves_, EpoR in Epo-Cy5.5 vesicles; Epo-Cy5.5_mem_, Epo-Cy5.5 bound to membrane EpoR; Epo-Cy5.5_cpl_, cytosolic Epo-Cy5.5). **(B)** Residuals shown as relative deviations between model fits and experimental data from bleached cells as in (A) (EpoR-GFP_mem_, membrane EpoR; EpoR-GFP_ves_, Epo in vesicles). **(C)** Residuals shown as relative deviations between model fits and experimental data from CHX-treated cells as in (A).
