# Supplementary material for: Correlated receptor transport processes buffer single-cell heterogeneity
Source: PLoS Comput Biol. 2017 Sep 25;13(9):e1005779. doi: 10.1371/journal.pcbi.1005779 (PMC5659801; doi:10.1371/journal.pcbi.1005779)
Supplement: S6 Fig — (DOCX) [file pcbi.1005779.s008.docx]

**
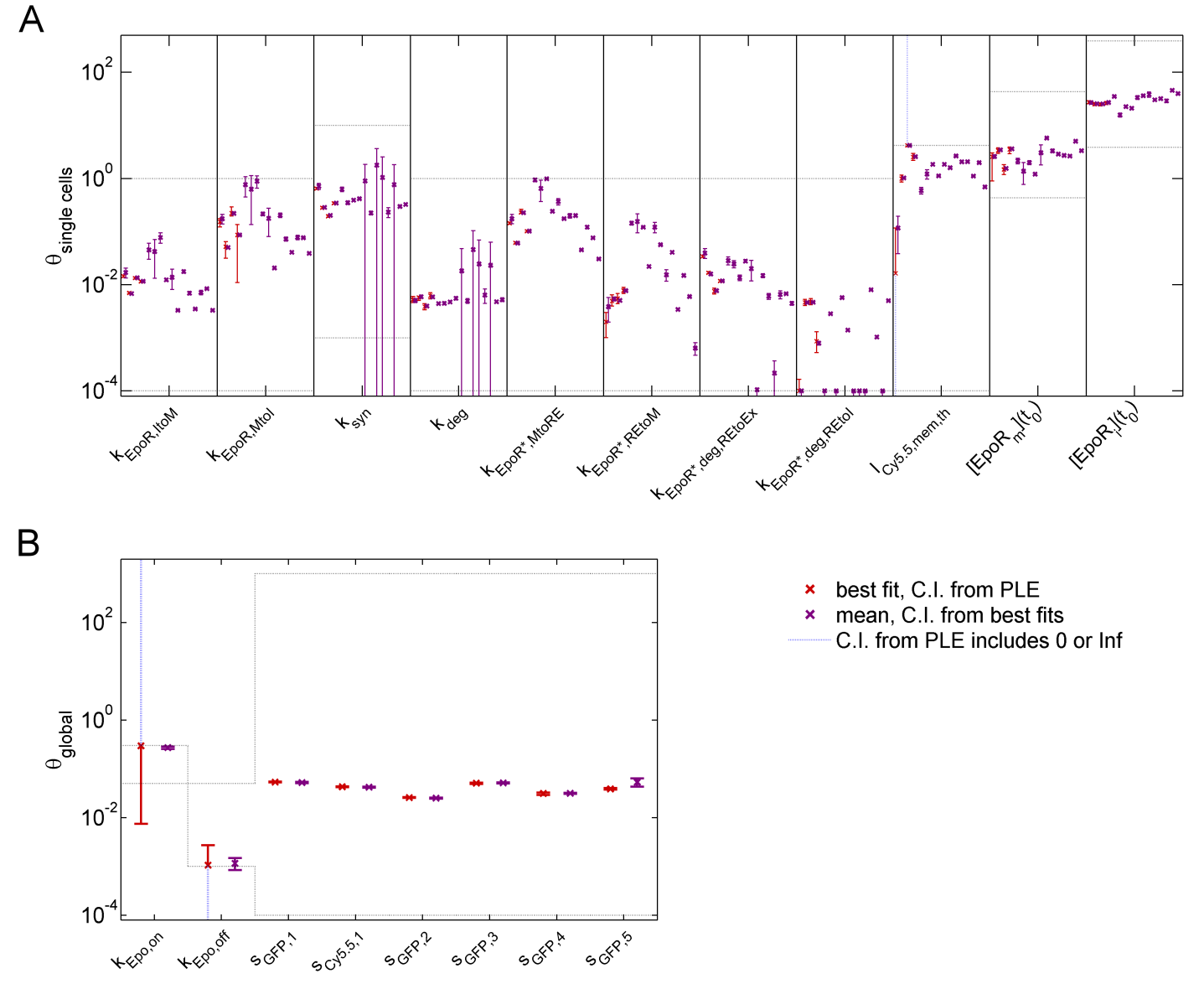
**

**S6 Fig.** **Parameter estimates for Epo-Cy5.5 internalizing cells from fitting variant ACD to the complete dataset.** **(A)** Single-cell parameter estimates from model fits to the complete dataset comprising trajectories of Epo-Cy5.5-internalizing (n=16), bleached (n=10) or CHX treated cells (n=7). Best fits and 1σ-confidence intervals from profile likelihood estimation are indicated for a subset of four cells as in Fig 4B and in S6 Table. For all parameters, mean parameters and from the best 0.5% of 1000 fits and 1σ-confidence intervals are indicated (k_EpoR,ItoM_, k_EpoR,MtoI_, transport of free EpoR between intracellular and plasma membrane compartments; k_EpoR,syn_, k_EpoR,deg_, EpoR synthesis and degradation; k_EpoR*,MtoRE_, endocytosis of Epo-ligated EpoR; k_EpoR*,REtoM_, EpoR transport back to plasma membrane; k_EpoR*,deg,REtoEx_, EpoR degradation with exocytosis of degraded Epo; k_EpoR*,deg,REtoI_, EpoR degradation with intracellular accumulation of degraded Epo; I_Cy5.5,mem,th_, Cy5.5 fluorescence intensity threshold in the plasma membrane ROI as defined in S2 Text, Eq. 6; [EpoR_m_](t_0_), EpoR concentration in the plasma membrane compartment at the start of the experiment; [EpoR_i_](t_0_), EpoR concentration in the intracellular compartment at the start of the experiment; C. I., confidence interval; PLE, profile likelihood estimation; dotted grey vertical lines, allowed intervals for estimated parameters). **(B)** Global parameter estimates as in (A) (k_on,Epo_, k_off,Epo_, Epo binding and unbinding; s_GFP,1_ to s_GFP,5_, s_Cy5.5,1_, scaling factors for GFP fluorescence and Cy5.5 fluorescence between experimental measurements in arbitrary units and model variables from different experiments).
