# Supplementary material for: Correlated receptor transport processes buffer single-cell heterogeneity
Source: PLoS Comput Biol. 2017 Sep 25;13(9):e1005779. doi: 10.1371/journal.pcbi.1005779 (PMC5659801; doi:10.1371/journal.pcbi.1005779)
Supplement: S9 Fig — (DOCX) [file pcbi.1005779.s011.docx]

**
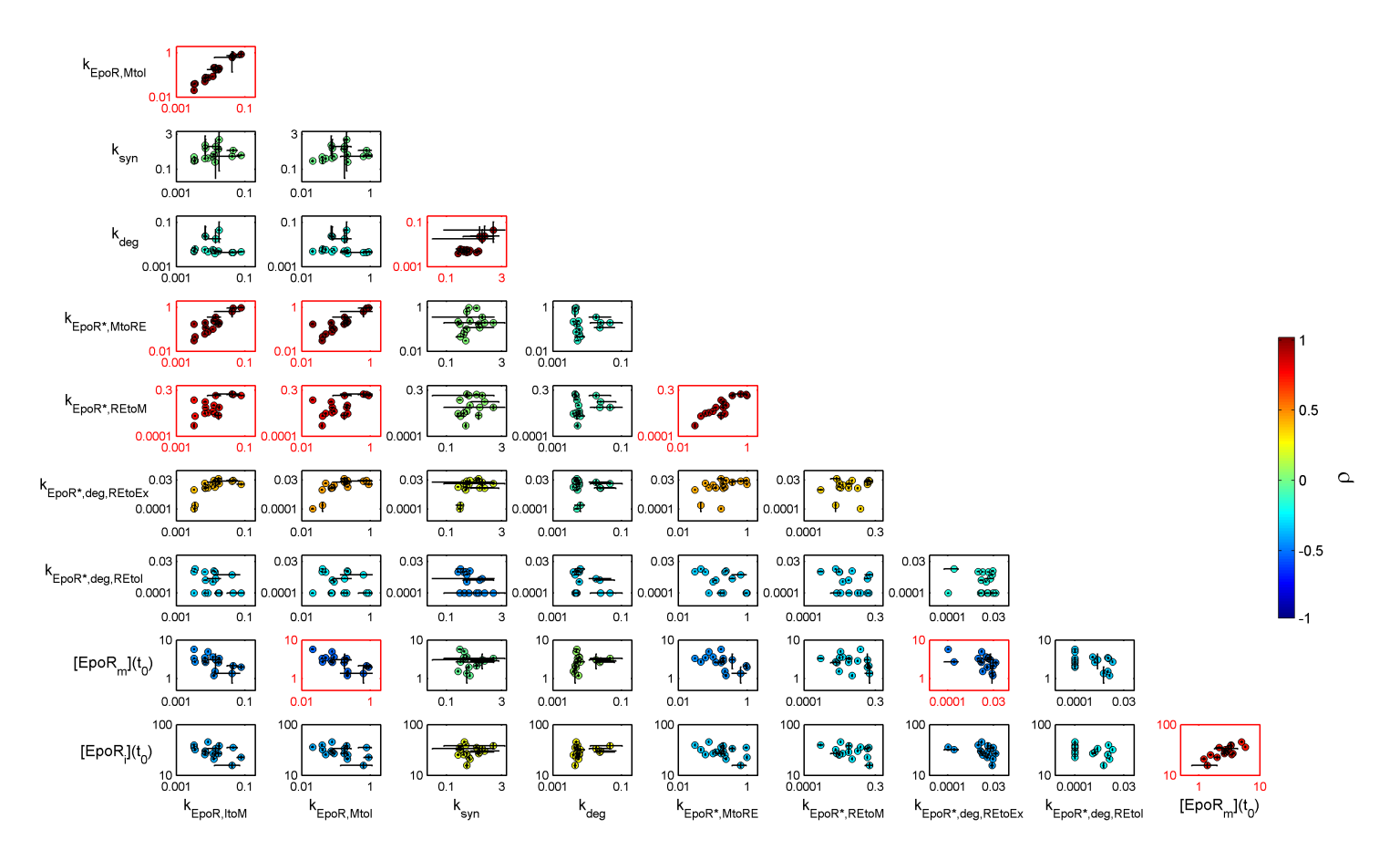
**

**S9 Fig.** **Correlations between single-cell parameter estimates.** For all parameters, mean parameters from the best 0.5% of 1000 fits of the variant ACD are shown on logarithmic scales with 1σ-confidence intervals indicated by error bars. Circle colors indicate the values of correlation coefficients and red boxes indicate significant correlation with p<0.05 (ρ, Pearson correlation coefficient).
