# Supplementary material for: Correlated receptor transport processes buffer single-cell heterogeneity
Source: PLoS Comput Biol. 2017 Sep 25;13(9):e1005779. doi: 10.1371/journal.pcbi.1005779 (PMC5659801; doi:10.1371/journal.pcbi.1005779)
Supplement: S1 Table — Reaction rates for variants of the EpoR traffic model with variable parts A to D. (DOCX) [file pcbi.1005779.s012.docx]

**S1 Table.** Reaction rates for variants of the EpoR traffic model with variable parts A to D.

| Reaction rates,  basic model | Reaction rates,  variable parts A to D | Comment |
| --- | --- | --- |
|  |  |  |
|  |  | Direct recycling to plasma membrane |
|  |  | Recycling to intracellular pool EpoR |
|  |  | Degradation with exocytosis of internalized Epo |
|  |  | Degradation with intracellular accumulation of internalized Epo |
|  |  |  |
|  |  |  |
