# Supplementary material for: Correlated receptor transport processes buffer single-cell heterogeneity
Source: PLoS Comput Biol. 2017 Sep 25;13(9):e1005779. doi: 10.1371/journal.pcbi.1005779 (PMC5659801; doi:10.1371/journal.pcbi.1005779)
Supplement: S2 Table — (DOCX) [file pcbi.1005779.s013.docx]

**S2 Table.** Equations of the EpoR traffic model variants. For each variant the variable terms for A to D were included or set to zero.

| Differential equations |  |  |  |  |
| --- | --- | --- | --- | --- |
| Basic model | A | B | C | D |
|  |  |  |  |  |
|  |  |  |  |  |
|  |  |  |  |  |
|  |  |  |  |  |
|  |  |  |  |  |
|  |  |  |  |  |
