# Supplementary material for: Correlated receptor transport processes buffer single-cell heterogeneity
Source: PLoS Comput Biol. 2017 Sep 25;13(9):e1005779. doi: 10.1371/journal.pcbi.1005779 (PMC5659801; doi:10.1371/journal.pcbi.1005779)
Supplement: S3 Table — (DOCX) [file pcbi.1005779.s014.docx]

**S3 Table.** Links between observables and model variables.

| Observable | Comment |
| --- | --- |
|  | Total amount of membrane bound EpoR-GFP |
|  | Intracellular EpoR-GFP |
|  | Intracellular EpoR-GFP colocalized with Cy5.5 |
|  | Epo-Cy5.5 bound to membrane EpoR-GFP |
|  | Intracellular Epo-Cy5.5, associated with EpoR-GFP or degraded |
