# Supplementary material for: Correlated receptor transport processes buffer single-cell heterogeneity
Source: PLoS Comput Biol. 2017 Sep 25;13(9):e1005779. doi: 10.1371/journal.pcbi.1005779 (PMC5659801; doi:10.1371/journal.pcbi.1005779)
Supplement: S4 Table — (DOCX) [file pcbi.1005779.s015.docx]

**S4 Table.** Reaction rates for auxiliary EpoR traffic models.

| Reaction rates |
| --- |
|  |
|  |
|  |
|  |
|  |

denotes the Heaviside step function, which is zero for and equals for .
