# Supplementary material for: Correlated receptor transport processes buffer single-cell heterogeneity
Source: PLoS Comput Biol. 2017 Sep 25;13(9):e1005779. doi: 10.1371/journal.pcbi.1005779 (PMC5659801; doi:10.1371/journal.pcbi.1005779)
Supplement: S5 Table — (DOCX) [file pcbi.1005779.s016.docx]

**S5 Table.** Equations of the auxiliary EpoR traffic models.

| Differential equations, auxiliary model |  |
| --- | --- |
| Photobleached cells | Cycloheximide (CHX) treated cells |
|  |  |
|  |  |
|  |  |
