# Supplementary material for: Correlated receptor transport processes buffer single-cell heterogeneity
Source: PLoS Comput Biol. 2017 Sep 25;13(9):e1005779. doi: 10.1371/journal.pcbi.1005779 (PMC5659801; doi:10.1371/journal.pcbi.1005779)
Supplement: S6 Table — (DOCX) [file pcbi.1005779.s017.docx]

**S6 Table.** Global parameter and single-cell parameter estimates as shown in Fig 4.

| Parameter | Best fit (C.I. from PLE) (C.I. in %) | Mean, SD from best fits (SD in %) |
| --- | --- | --- |
|  in 1/(nM·min) | 0.2962 (0.2887, Inf) (-2.5%, Inf) | 0.2725 ±0.0154 (±5.6%) |
|  in 1/nM | 0.0011 (0, 0.0038) (-100.0%, 255.6%) | 0.0012 ±0.0003 (±27.7%) |
|  in 1/nM | 0.0535 (0.0525, 0.0546) (-1.9%, 1.9%) | 0.0525 ±0.0014 (±2.7%) |
|  in 1/nM | 0.0430 (0.0422, 0.0438) (-1.9%, 1.9%) | 0.0422 ±0.0010 (±2.4%) |
|  in 1/nM | 0.0258 (0.0253, 0.0263) (-1.9%, 1.9%) | 0.0251 ±0.0008 (±3.1%) |
|  in 1/nM | 0.0509 (0.0490, 0.0519) (-3.8%, 1.9%) | 0.0515 ±0.0013 (±2.5%) |
|  in 1/nM | 0.0313 (0.0290, 0.0326) (-7.4%, 3.9%) | 0.0315 ±0.0007 (±2.4%) |
|  in 1/nM | 0.0389 (0.0375, 0.0404) (-3.8%, 3.9%) | 0.0534 ±0.0105 (±19.6%) |
|  in 1/min | 0.0144 (0.0138, 0.0151) (-4.3%, 4.5%) | 0.0170 ±0.0036 (±21.2%) |
|  in 1/min | 0.0070 (0.0067, 0.0073) (-4.3%, 4.5%) | 0.0068 ±0.0003 (±4.4%) |
|  in 1/min | 0.0133 (0.0127, 0.0139) (-4.3%, 4.5%) | 0.0135 ±0.0004 (±2.8%) |
|  in 1/min | 0.0115 (0.0110, 0.0120) (-4.3%, 4.5%) | 0.0116 ±0.0002 (±2.0%) |
|  in 1/min | 0.1532 (0.1225, 0.1747) (-20.0%, 14.1%) | 0.1745 ±0.0359 (±20.6%) |
|  in 1/min | 0.0519 (0.0316, 0.0649) (-39.1%, 25.1%) | 0.0501 ±0.0026 (±5.1%) |
|  in 1/min | 0.2233 (0.1957, 0.2917) (-12.3%, 30.7%) | 0.2203 ±0.0101 (±4.6%) |
|  in 1/min | 0.0865 (0.0110, 0.1358) (-87.3%, 57.1%) | 0.0866 ±0.0020 (±2.3%) |
|  in nM/min | 0.6470 (0.6192, 0.6760) (-4.3%, 4.5%) | 0.7141 ±0.0943 (±13.2%) |
|  in nM/min | 0.2818 (0.2697, 0.2944) (-4.3%, 4.5%) | 0.2854 ±0.0142 (±5.0%) |
|  in nM/min | 0.1945 (0.1861, 0.2032) (-4.3%, 4.5%) | 0.2026 ±0.0093 (±4.6%) |
|  in nM/min | 0.3425 (0.3278, 0.3578) (-4.3%, 4.5%) | 0.3452 ±0.0086 (±2.5%) |
|  in 1/min | 0.0053 (0.0046, 0.0060) (-12.3%, 14.1%) | 0.0050 ±0.0005 (±9.2%) |
|  in 1/min | 0.0057 (0.0050, 0.0060) (-12.3%, 4.5%) | 0.0059 ±0.0004 (±6.6%) |
|  in 1/min | 0.0038 (0.0034, 0.0044) (-12.3%, 14.1%) | 0.0040 ±0.0002 (±4.1%) |
|  in 1/min | 0.0062 (0.0054, 0.0071) (-12.3%, 14.1%) | 0.0059 ±0.0004 (±6.1%) |
|  in 1/min | 0.1439 (0.1378, 0.1504) (-4.3%, 4.5%) | 0.1747 ±0.0350 (±20.0%) |
|  in 1/min | 0.0614 (0.0588, 0.0642) (-4.3%, 4.5%) | 0.0608 ±0.0012 (±1.9%) |
|  in 1/min | 0.2293 (0.2194, 0.2615) (-4.3%, 14.1%) | 0.2276 ±0.0044 (±1.9%) |
|  in 1/min | 0.1020 (0.0976, 0.1066) (-4.3%, 4.5%) | 0.1024 ±0.0011 (±1.1%) |
|  in 1/min | 0.0020 (0.0010, 0.0030) (-49.6%, 50.4%) | 0.0039 ±0.0019 (±48.7%) |
|  in 1/min | 0.0049 (0.0039, 0.0064) (-20.0%, 30.7%) | 0.0054 ±0.0005 (±8.8%) |
|  in 1/min | 0.0055 (0.0044, 0.0069) (-20.0%, 25.1%) | 0.0050 ±0.0003 (±6.2%) |
|  in 1/min | 0.0078 (0.0068, 0.0089) (-12.3%, 14.1%) | 0.0077 ±0.0001 (±0.8%) |
|  in 1/min | 0.0341 (0.0326, 0.0356) (-4.3%, 4.5%) | 0.0393 ±0.0082 (±21.0%) |
|  in 1/min | 0.0168 (0.0161, 0.0176) (-4.3%, 4.5%) | 0.0159 ±0.0011 (±6.6%) |
|  in 1/min | 0.0076 (0.0066, 0.0086) (-12.3%, 14.1%) | 0.0077 ±0.0003 (±4.5%) |
|  in 1/min | 0.0117 (0.0112, 0.0123) (-4.3%, 4.5%) | 0.0118 ±0.0003 (±2.3%) |
|  in 1/min | 0.0001 (0, 0.0002) (-100.0%, 64.9%) | 0.0001 ±0.000001 (±0.7%) |
|  in 1/min | 0.0046 (0.0041, 0.0053) (-12.3%, 14.1%) | 0.0046 ±0.00005 (±1.0%) |
|  in 1/min | 0.0048 (0.0042, 0.0054) (-12.3%, 14.1%) | 0.0047 ±0.0001 (±1.9%) |
|  in 1/min | 0.0009 (0.0005, 0.0013) (-39.3%, 50.4%) | 0.0008 ±0.0001 (±7.9%) |
|  in nM | 0.0164 (0, 0.1164) (-100.0%, 611.2%) | 0.1167 ±0.0783 (±67.1%) |
|  in nM | 0.9964 (0.8558, 1.1600) (-14.1%, 16.4%) | 1.0258 ±0.0215 (±2.1%) |
|  in nM | 4.2000 (3.9924, Inf) (-4.9%, Inf) | 4.2000 ±0.0001 (±0.0%) |
|  in nM | 2.5438 (2.1850, 2.9617) (-14.1%, 16.4%) | 2.5785 ±0.0487 (±1.9%) |
|  in nM | 2.5751 (0.9007, 3.0155) (-65.0%, 17.1%) | 2.6030 ±0.1942 (±7.5%) |
|  in nM | 3.3690 (2.9472, 3.7676) (-12.5%, 11.8%) | 3.4686 ±0.1065 (±3.1%) |
|  in nM | 1.4822 (1.1825, 1.8175) (-20.2%, 22.6%) | 1.5542 ±0.0754 (±4.9%) |
|  in nM | 3.5102 (2.9325, 3.9256) (-16.5%, 11.8%) | 3.6120 ±0.1308 (±3.6%) |
|  in nM | 27.3439 (25.6029, 29.2033) (-6.4%, 6.8%) | 26.8438 ±1.9172 (±7.1%) |
|  in nM | 25.0555 (23.4602, 26.7593) (-6.4%, 6.8%) | 25.7008 ±0.7636 (±3.0%) |
|  in nM | 24.8972 (23.3119, 26.5902) (-6.4%, 6.8%) | 25.4251 ±0.8018 (±3.2%) |
|  in nM | 26.4069 (24.7255, 28.2026) (-6.4%, 6.8%) | 27.0155 ±0.8743 (±3.2%) |
