# Supplementary material for: Correlated receptor transport processes buffer single-cell heterogeneity
Source: PLoS Comput Biol. 2017 Sep 25;13(9):e1005779. doi: 10.1371/journal.pcbi.1005779 (PMC5659801; doi:10.1371/journal.pcbi.1005779)
Supplement: S7 Table — (DOCX) [file pcbi.1005779.s018.docx]

**S7 Table.** Single-cell log-normal parameter distributions.

| Parameter | Mean (1σ-C.I.)* | µ | σ |
| --- | --- | --- | --- |
|  in 1/min | 0.0181 (0.0046, 0.0297) | -4.4472 | 0.9306 |
|  in 1/min | 0.2337 (0.0434, 0.3974) | -2.0298 | 1.1069 |
|  in nM/min | 0.5569 (0.2427, 0.8509) | -0.7887 | 0.6272 |
|  in 1/min | 0.0108 (0.0035, 0.0165) | -4.8739 | 0.7671 |
|  in 1/min | 0.2877 (0.0654, 0.4953) | -1.7153 | 1.0127 |
|  in 1/min | 0.0452 (0.0033, 0.0852) | -4.0853 | 1.6228 |
|  in 1/min | 0.0143 (0.0014, 0.0400) | -4.8801 | 1.6602 |
|  in 1/min | 0.0022 (0.0001, 0.0042) | -7.3080 | 1.8336 |

*Distribution parameters (means, 1σ-confidence intervals for parameter distributions, µ and σ) were estimated by fitting log-normal distribution functions to single-cell parameter estimates.
